# Computational evaluation of aluminum and zinc doped C_20_ fullerenes as advanced sensors for the detection of the narcotic dimethyltryptamine

**DOI:** 10.1038/s41598-026-41537-9

**Published:** 2026-03-09

**Authors:** Saad M. Alshahrani

**Affiliations:** Department of Pharmaceutics, College of Pharmacy, Prince Sattam Bin Abdulaziz University, P.O. Box 173, Al-Kharj, 11942 Saudi Arabia

**Keywords:** Adsorption, Colorimetric sensors, Electrochemical sensor, dimethyltryptamine, DFT, NCI, Chemistry, Environmental sciences, Materials science, Nanoscience and technology

## Abstract

N, N-Dimethyltryptamine (N, N-DMT) is a potent psychedelic substance whose detection is crucial in medical and forensic contexts. In this study, we computationally evaluate the potential of aluminum- and zinc-doped C_20_ fullerenes (AlC_19_ and ZnC_19_) as advanced sensors for N, N-DMT detection. Using density functional theory (DFT) and time-dependent DFT, along with NBO, NCI, RDG, and ESP analyses, we assess key sensing parameters including adsorption energy, recovery time, electrical conductivity, and UV-vis spectral shifts. Results reveal that AlC_19_ exhibits the strongest adsorption energy (-49.57 kcal/mol), making it suitable for N, N-DMT capture and removal. In contrast, ZnC_19_ shows a significant conductivity decrease upon adsorption and a pronounced redshift in absorption wavelength (from 455 nm to 523 nm), along with a practical recovery time (~ 3.70 × 10⁴ s). These features make ZnC_19_ a highly promising candidate for real-time electrochemical and colorimetric sensing of N, N-DMT, while AlC_19_ is better suited for adsorption applications.

## Introduction

N, N-Dimethyltryptamine (N, N-DMT) is a potent psychoactive indole alkaloid encountered in clinical toxicology, forensic investigations, and public-health monitoring due to its rapid pharmacological action and increasing non-medical use. Because N, N-DMT is often obtained from unregulated sources and may be present at trace levels or in complex matrices, its reliable identification is critical for medical decision-making, forensic confirmation, and harm-reduction efforts. These factors emphasize the need for rapid, sensitive, and reliable diagnostic strategies^1,2^.

Detecting N, N-DMT is commonly performed using methods such as gas chromatography-mass spectrometry (GC-MS), liquid chromatography-mass spectrometry (LC-MS), and immunoassay-based techniques. These methods require expensive instrumentation, skilled operators, and time-consuming sample preparation. Most importantly, none of them are always available. Given these limitations, an urgent need is felt for a rapid, cost-effective, and accessible method for N, N-DMT detection. In this context, the use of nanomaterials as electrochemical sensors has recently attracted significant attention from researchers^3–5^. Nanomaterials such as carbon nanotubes (CNTs), graphene, metal nanoparticles, and quantum dots have shown great potential in enhancing the sensitivity and selectivity of drug detection systems. Among these, fullerenes, particularly fullerene C_20_, are of special interest due to their unique structural and electronic properties^6–8^. Fullerene C_20_ offers several advantages as an electrochemical sensor, including a high surface area, excellent electron transfer capabilities, and strong chemical stability, which enhance its sensitivity in detecting trace amounts of drugs like N, N- N, N-DMT. Additionally, its small size and unique curvature contribute to increased interactions with target molecules, improving detection efficiency^9–11^.

The role of C_20_ as an electrochemical sensor has recently been explored in various scientific literatures, highlighting its potential in drug analysis and forensic applications. For example, recently, W. Zhao et al. proposed fullerene (C_20_) as an electrochemical sensor for the detection of NO, CO, and O_2_, and demonstrated its high sensitivity and selectivity^12^. In another study, B. Ajdari et al. investigated the capability of fullerene (C_20_) as a sensing material for the electrochemical detection of nortriptyline, highlighting its efficient electron transfer properties^13^. Additionally, N. Khalaj Zeighami et al. explored the dual role of fullerene (C_20_) as both a sensor and adsorbent for aflatoxin, emphasizing its strong adsorption capacity and stability^14^. Furthermore, P. Niknam Rad et al. proposed fullerene (C_20_) as an electrochemical sensor for the detection of perphenazine, showcasing its rapid response and excellent detection performance^15^. These studies collectively underline the growing interest in fullerene (C_20_) as a versatile and effective material for electrochemical sensing applications. As research progresses, fullerene-based sensors may offer a breakthrough in developing rapid and reliable detection systems for psychoactive substances like DMT.

Given the emphasis in various literatures on the use of C_20_ fullerene in the design of electrochemical sensors and the critical need for rapid and efficient N, N-DMT detection, we aim to develop a new generation of electrochemical sensors based on pristine C_20_ fullerene doped with aluminum and zinc. The doping of C_20_ with aluminum and zinc is intended to enhance its electronic properties, such as conductivity, charge transfer efficiency, and sensitivity, which are crucial for improving the detection capabilities of the sensor. As emphasized in various literatures, aluminum doping can enhance electron-donating properties, while zinc can increase electron mobility and stability, both of which contribute to more efficient interactions with N, N-DMT molecules^16–19^. While fullerene C_20_ has been employed in a variety of sensor applications, particularly for detecting gases and other organic molecules, its use in detecting N, N-Dimethyltryptamine (N, N-DMT) has not been investigated in the context of electrochemical or colorimetric sensing. Notably, previous studies have explored C_20_ and its derivatives for detecting molecules such as nitric oxide, perphenazine, and aflatoxin. However, this work represents the first systematic exploration of C_20_ and its doped forms, specifically AlC_19_ and ZnC_19_, for the detection of N, N-DMT, a potent psychoactive substance. The unique combination of C_20_-based systems and N, N-DMT detection, as well as the application of computational methods to optimize these sensor systems, provides a novel approach that has not been extensively studied or reported in the existing literature. This study aims to fill this gap by demonstrating the potential of C_20_-based systems as advanced sensors for N, N-DMT detection, offering insights into their use in forensic science, healthcare, and drug monitoring.

To investigate the feasibility of achieving the goals of this study, we used computational chemistry as an efficient initial strategy prior to material synthesis. Using theoretical methods prior to experimental work offers several advantages: it reduces costs and time by predicting molecular interactions in advance, helps identify the most promising structures without the need for extensive trial-and-error, and provides detailed insight into electronic behavior that may be difficult to obtain experimentally. In this study, we applied density functional theory (DFT) to analyze structural and electronic properties and, time-dependent DFT (TD-DFT) to explore the optical and excitation features relevant to sensing performance. These computational approaches allowed us to efficiently evaluate the potential of the C_20_-based systems for detecting N, N-DMT before moving to laboratory synthesis^20–23^. We hope that the results of this research will contribute to the development of advanced and colorimetric sensors, proving useful in the rapid and accurate detection of N, N-DMT, with potential applications in forensic science, healthcare, and drug monitoring.

While pristine C_20_ fullerene exhibits promising sensing properties, its practical application is often limited by moderate sensitivity, selectivity, and recovery characteristics. In this work, we introduce Al- and Zn-doped C_19_ fullerenes as novel sensor materials with tailored electronic and structural properties that address these limitations. Doping with aluminum enhances electron-donating capacity and strong adsorption, making AlC_19_ particularly suitable for irreversible capture and removal of N, N-DMT. In contrast, zinc doping improves electron mobility and modulates the HOMO-LUMO gap more effectively, leading to significant changes in electrical conductivity and optical absorption upon analyte binding. These doped systems not only surpass pristine C_20_ in terms of sensitivity and selectivity but also offer distinct functional advantages (AlC_19_ for adsorption and ZnC_19_ for reversible electrochemical and colorimetric detection). This dual-functional approach, enabled by targeted doping, represents a significant advancement in the design of fullerene-based sensors for psychoactive substances.

## Calculation section

**Fig. 1:**
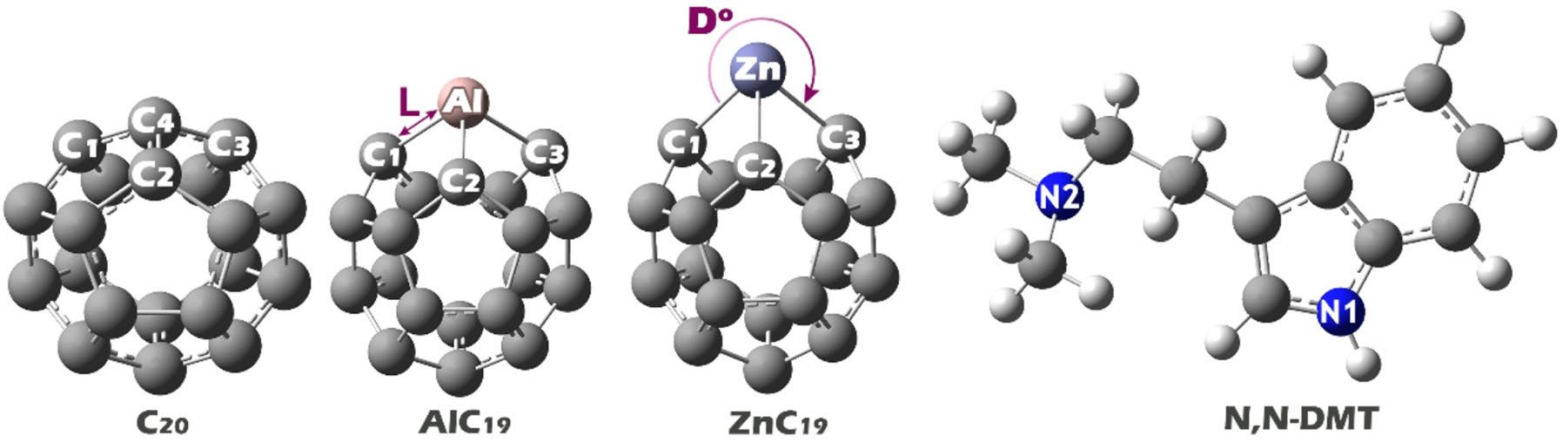
Optimized molecular structures of N, N-DMT, pristine C_20_ fullerene, AlC_19_, and, ZnC_19_, as obtained from DFT geometry optimization at the B3LYP/6-311G(d, p) level in aqueous phase (CPCM model).

All the structures (N, N-DMT, C_20_, AlC_19_, and ZnC_19_) in this study were designed using GaussView software. Afterward, their geometric structures were optimized using Gaussian software (Fig. 1)^24,25^. The geometric structure optimization was carried out using density functional theory (DFT) at the B3LYP/6-311G(d, p) computational level. The B3LYP functional was selected due to its proven accuracy and reliability in predicting geometric, electronic, and energetic properties of carbon-based nanostructures and organic molecules, as evidenced by numerous benchmark studies in the literature. The 6-311G(d, p) basis set provides a balanced description of both core and valence electrons, offering a good compromise between computational cost and accuracy for systems of this size and composition. This level of theory has been successfully applied in similar studies of fullerene-based sensors and adsorption systems, ensuring consistency and comparability with existing computational work^26,27^. Also, the self-consistent field (SCF) calculations employed the following convergence thresholds during geometry optimization: the root-mean-square (RMS) density matrix change was required to be less than 1.00 × 10^− 8^, and the maximum density matrix change was required to be less than 1.00 × 10^− 6^. These stringent criteria ensured robust convergence of the electronic wavefunction at each optimization step. For the final optimized geometry, the SCF energy convergence reached an RMS density change of 0.41 × 10^− 8^, satisfying both conditions and confirming a fully converged electronic structure. All calculations were performed in the aqueous phase using the Continuous Polarizable Charge Model (CPCM), which simulates the solvation effect by incorporating the solvent (water, in this case). The choice of water as the environment for these calculations is based on the fact that many chemical interactions, particularly in biorelevant systems such as drug detection, occur in aqueous environments, mimicking real-life conditions. This allows for more accurate predictions of the behavior of the designed sensors in practical applications^28,29^.

Frequency calculations were also conducted at the same theoretical level to confirm the stability of the optimized structures. The absence of imaginary frequencies indicates that the designed structures are stable and not in a transition state, further validating the feasibility of these structures as viable electrochemical sensors for N, N-DMT detection^30^.

The stability of the designed systems was evaluated through cohesive energy calculations, as defined in Eq. (1).

1 $$\:{\boldsymbol{E}}_{\boldsymbol{C}\boldsymbol{o}\boldsymbol{h}}=-({\boldsymbol{E}}_{\boldsymbol{t}\boldsymbol{o}\boldsymbol{t}}-\sum\:_{\boldsymbol{i}}{\boldsymbol{n}}_{\boldsymbol{i}}{\boldsymbol{E}}_{\boldsymbol{i}})/\boldsymbol{n}$$

E_tot_: Total electronic energy of the optimized molecule or complex. ∑*n*_i_*E*_i_: The summation of the total energies of the isolated atoms making up the structure. Here, *E*_i_ is the energy of the atom, and *ni* is the number of atoms of that type in the structure. *n*: Total number of atoms in the structure^31^.

The electronic and quantum properties of the designed structures were calculated using key descriptors, including energy gap (HLG), chemical softness (S), chemical hardness (η), chemical potential (µ), maximum charge transfer value (ΔNmax), and electrophilicity-based charge transfer (ECT), according to Eqs. (2) to (7), respectively.

2 $$\:\mathbf{H}\mathbf{L}\mathbf{G}=\left|{\mathbf{E}}_{\mathbf{H}\mathbf{O}\mathbf{M}\mathbf{O}}-{\mathbf{E}}_{\mathbf{L}\mathbf{U}\mathbf{M}\mathbf{O}}\right|$$

3 $$\:\boldsymbol{\upeta\:}=\raisebox{1ex}{$(-{\mathbf{E}}_{\mathbf{H}\mathbf{O}\mathbf{M}\mathbf{O}}-(-{\mathbf{E}}_{\mathbf{L}\mathbf{U}\mathbf{M}\mathbf{O}}\:\left)\right)$}\!\left/\:\!\raisebox{-1ex}{$2$}\right.$$

4 $$\:\boldsymbol{\upmu\:}=-(-{\mathbf{E}}_{\mathbf{H}\mathbf{O}\mathbf{M}\mathbf{O}}+(-{\mathbf{E}}_{\mathbf{L}\mathbf{U}\mathbf{M}\mathbf{O}}\left)\right)/2$$

5 $$\:\boldsymbol{S}=1/2\boldsymbol{\upeta\:}$$

6 $$\:{\varDelta\:\boldsymbol{N}}_{\boldsymbol{m}\boldsymbol{a}\boldsymbol{x}}=-\raisebox{1ex}{$\boldsymbol{\mu\:}$}\!\left/\:\!\raisebox{-1ex}{$\boldsymbol{\eta\:}$}\right.$$

7 $$\:\boldsymbol{E}\boldsymbol{C}\boldsymbol{T}={\left({\boldsymbol{\Delta\:}\boldsymbol{N}}_{\boldsymbol{m}\boldsymbol{a}\boldsymbol{x}}\right)}_{\boldsymbol{\alpha\:}}-{\left({\boldsymbol{\Delta\:}\boldsymbol{N}}_{\boldsymbol{m}\boldsymbol{a}\boldsymbol{x}}\right)}_{\boldsymbol{\beta\:}}$$

E_HOMO_ and E_LUMO_ represent the energies of the highest occupied (HOMO) and lowest unoccupied (LUMO) molecular orbitals, respectively, and are key indicators of a molecule’s electron-donating and -accepting abilities^32–34^.

ΔN_max_ represents the maximum charge transfer, with α for the complex and β for the (Al/Zn)-doped C_20_. A positive ECT means the sensor donates electrons to N, N-DMT, while a negative ECT indicates electron transfer from N, N-DMT to the sensor^35,36^.

The sensing mechanism of each designed structure was calculated and evaluated using key parameters including adsorption energy (Eads), recovery time (τ), and electrical conductivity (σ) (Eqs. 8–10).

8 $$\:{\mathbf{E}}_{\mathbf{a}\mathbf{d}\mathbf{s}}={\mathbf{E}}_{\left(\mathbf{R}-\right)\mathbf{C}20@\mathbf{N},\mathbf{N}-\mathbf{D}\mathbf{M}\mathbf{T}}-\left({\mathbf{E}}_{\boldsymbol{N},\boldsymbol{N}-\boldsymbol{D}\boldsymbol{M}\boldsymbol{T}}+{\mathbf{E}}_{\left(\mathbf{R}-\right)\mathbf{C}20}\right)$$

9 $$\:\boldsymbol{\tau\:}={\boldsymbol{V}}_{0}^{-1}\times\:\mathbf{e}\mathbf{x}\mathbf{p}(-\frac{{\boldsymbol{E}}_{\boldsymbol{a}\boldsymbol{d}\boldsymbol{s}}}{{\boldsymbol{k}}_{\boldsymbol{B}}\boldsymbol{T}})$$

10 $$\:\boldsymbol{\upsigma\:}=\boldsymbol{A}{\boldsymbol{T}}^{3/2}{\boldsymbol{e}}^{(-\boldsymbol{H}\boldsymbol{L}\boldsymbol{G}/2\boldsymbol{K}\boldsymbol{T})}$$

In Eq. (8), the adsorption energy (E_ads_) is computed using the total energy of the DMT–sensor complex (E_(R−)C20@N, N−DMT_), the energies of the isolated DMT (E_N, N−DMT_) and the sensor (E_(R−)C20_). Equation (9) defines the recovery time (τ) based on the adsorption energy, where V_0_ is the attempt frequency (typically ~ 10^12^ s^− 1^), kB is the Boltzmann constant, and T is the temperature (commonly 298 K). Lastly, Eq. (10) describes the electrical conductivity (σ), where A is the Richardson constant (6 × 10^5^ A.m^− 2^.K^− 2^), and T is the temperature (298 K)^37–39^.

And finally, the dipole moment was calculated using Eq. (11)^40^.

11 $$\:\boldsymbol{\mu\:}=\sqrt{{\boldsymbol{\mu\:}}_{\boldsymbol{x}}^{2}+{\boldsymbol{\mu\:}}_{\boldsymbol{y}}^{2}+{\boldsymbol{\mu\:}}_{\boldsymbol{z}}^{2}}$$

These calculations were systematically performed to investigate the adsorption behavior, electronic properties, and sensing potential of C_20_ fullerene and its functionalized derivatives for N, N-DMT detection, to contribute to the development of diagnostic tools.

## Results and discussion

### Structural properties

#### Bond length and bond angle

Studying bond length and bond angle is essential in the design of molecular structures, as these parameters directly influence the stability, geometry, and electronic properties of a molecule. When doping is introduced into a molecular structure, it can significantly alter these geometric parameters by introducing atoms with different sizes or electronic characteristics compared to the host atoms. Such modifications often lead to structural distortions, which in turn can impact the delocalization and mobility of π electrons (a critical factor in determining the electronic transport properties of the system). These distortions influence conjugation and electron density distribution, potentially enhancing or reducing the molecule’s conductivity and overall functionality^41–43^. In the present study, the calculated bond lengths and bond angles for key atoms are presented in Table 1. These values highlight the changes caused by impurity and indicate changes in bond geometry and electronic configuration.

**Table 1:** Calculated values ​​of bond lengths (L) and bond angles (D) between some important atoms in the designed structures.

Structure	Bond lengths (Å)		Bond angles (°)	
C_20_	C_4_-C_1_	1.44	C_1_-C_4_-C_2_	108.64
	C_4_-C_2_	1.44	C_1_-C_4_-C_3_	109.01
	C_4_-C_3_	1.44	C_2_-C_4_-C_3_	108.64
AlC_19_	Al-C_1_	1.93	C_1_-Al-C_2_	89.41
	Al-C_2_	1.93	C_1_-Al-C_3_	93.69
	Al-C_3_	1.94	C_2_-Al-C_3_	93.67
ZnC_19_	Zn-C_1_	2.06	C_1_-Zn-C_2_	81.69
	Zn-C_2_	2.06	C_1_-Zn-C_3_	81.64
	Zn-C_3_	2.06	C_2_-Zn-C_4_	81.63

Based on the calculated data presented in Table 1, notable differences in bond lengths and bond angles are observed among the undoped and doped structures, reflecting the influence of doping on molecular geometry. In the pristine C_20_ structure, the C-C bond lengths around the C_4_ atom are uniformly 1.44 Å, and the associated bond angles range from 108.64° to 109.01°, values that are consistent with typical sp^3^ hybridized carbon atoms in a stable, symmetric configuration. However, upon doping with aluminum in AlC_19_, significant changes occur. The Al-C bond lengths increase to approximately 1.93–1.94 Å, indicating a longer bond due to the larger atomic radius of aluminum compared to carbon. Additionally, the bond angles involving the Al atom deviate from the original structure, ranging from 89.41^°^ to 93.69^°^, suggesting a noticeable distortion in geometry.

In the ZnC_19_ structure, the effect of doping becomes even more pronounced. The Zn-C bond lengths further increase to 2.06 Å, consistent with the even larger atomic radius of zinc. Moreover, the bond angles around the Zn atom decrease sharply to approximately 81.63^°^-81.69^°^, indicating a significant compression of the local geometry. These structural distortions, caused by the introduction of Al and Zn dopants, confirm that doping alters the molecular framework by changing bond lengths and angles, which may consequently influence the delocalization and mobility of π-electrons in the system.

#### Cohesive energy

**Fig. 2:**
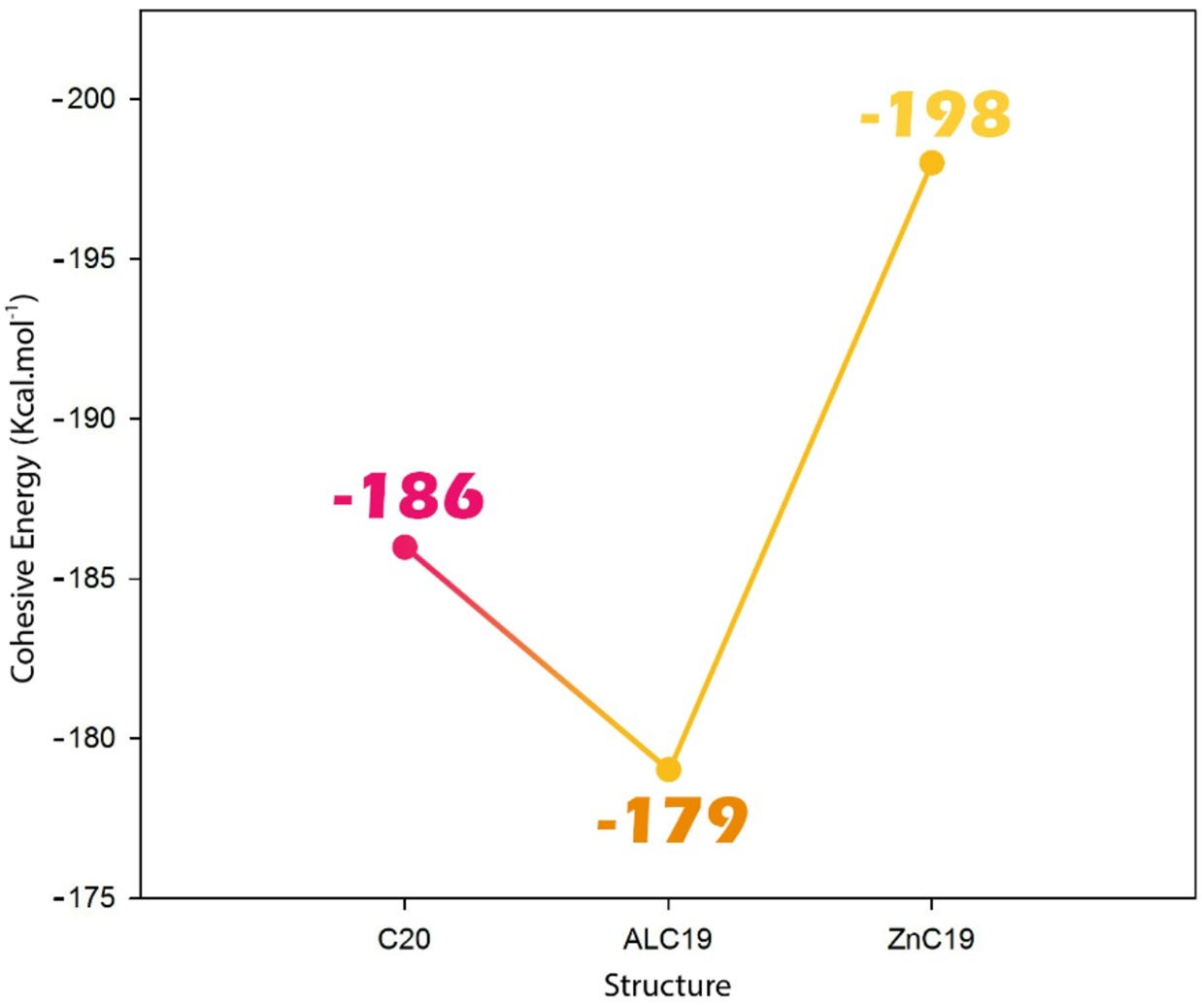
Cohesive energy (E_Coh_) values for pristine C_20_, AlC_19_, and ZnC_19_, indicating the effect of Al and Zn doping on structural stability. More negative values correspond to higher stability.

Cohesive energy is defined as the energy required to disassemble a compound into its individual isolated atoms, representing the strength of the interactions that hold the atoms together within a structure. It is a fundamental parameter for evaluating the thermodynamic stability of molecular and solid-state systems. A higher (more negative) cohesive energy generally indicates a more stable structure, as more energy is needed to break it apart^44,45^. In the design of molecular structures, considering cohesive energy is crucial because it provides insight into the overall stability and feasibility of the system. Studies show that doping atoms in molecular structures can significantly affect the coherence energy and strengthen or weaken atomic interactions in the host structure^46,47^. Therefore, to investigate the effect of impurity on the structural stability of each system, the coherence energy was calculated for all designed structures, and the results are reported in Fig. 2.

Based on the reported cohesive energy values, a clear variation in structural stability is observed among the undoped and doped systems. The pristine C_20_ structure exhibits a cohesive energy of -186 kcal/mol, indicating a relatively stable configuration. Upon doping with aluminum, the cohesive energy decreases to -179 kcal/mol, suggesting a reduction in structural stability. This reduction can be attributed to the incorporation of the Al atom, which introduces geometric distortions (reflected in the altered bond lengths and bond angles observed earlier) and weakens the overall atomic interactions within the structure.

In contrast, the ZnC_19_ structure shows an increased cohesive energy of -198 kcal/mol, indicating enhanced stability compared to both the undoped C_20_ and the Al-doped AlC_19_. Despite the significant compression in bond angles caused by Zn doping, the stronger interactions between Zn and surrounding carbon atoms appear to compensate for these distortions, resulting in a more tightly bound and energetically favorable configuration. These results are consistent with the previous observations, where structural distortions due to doping were shown to influence both geometry and stability. Thus, cohesive energy analysis confirms that while Al doping destabilizes the system, Zn doping enhances the structural integrity of the molecular framework.

### Electronic properties

#### Molecular electrostatic potential (MEP) contour

**Fig. 3:**
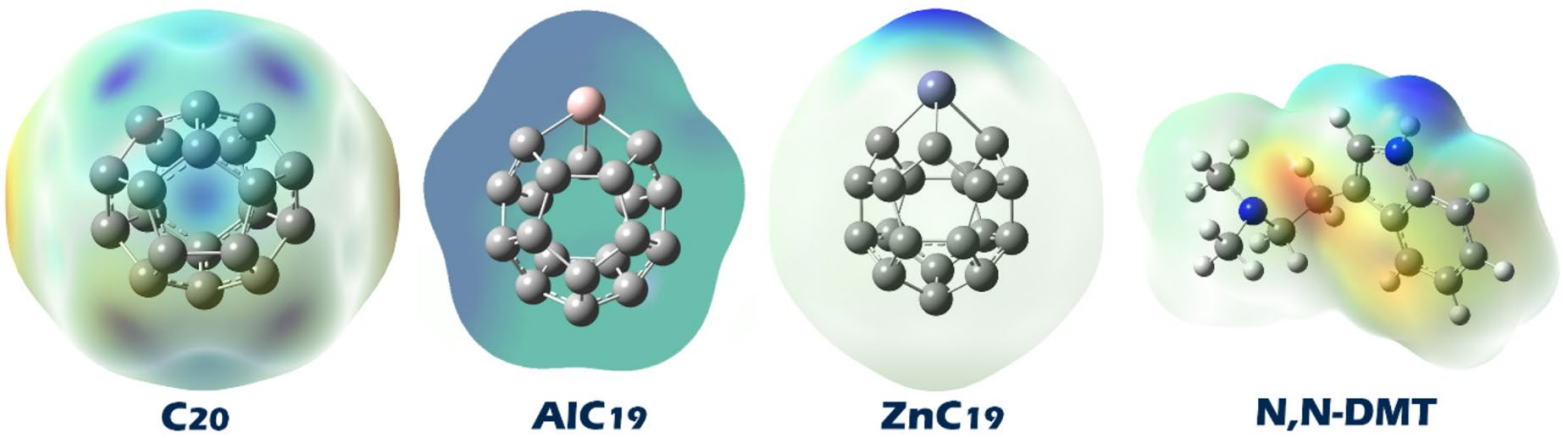
Molecular Electrostatic Potential (MEP) maps for N, N-DMT, C_20_, AlC_19_, and, ZnC_19_. Red regions indicate electron-rich areas (negative potential), blue regions indicate electron-deficient areas (positive potential), and green/yellow represent neutral zones.

Examining Molecular Electrostatic Potential (MEP) contours is crucial for identifying the most likely interaction points between molecules because MEP maps visually represent the distribution of electrostatic potential over a molecule’s surface. The color coding of these contours typically indicates regions of varying electron density and electrostatic charge, which directly relate to how molecules interact^48,49^. By analyzing these colored regions on the MEP map, the locations of favorable intermolecular interactions such as hydrogen bonding, electrostatic attractions, and reactive centers in chemical reactions can be predicted. Based on this, MEP maps were drawn for N, N-DMT, pristine C_20_, and its doped forms (Fig. 3).

**Fig. 4:**
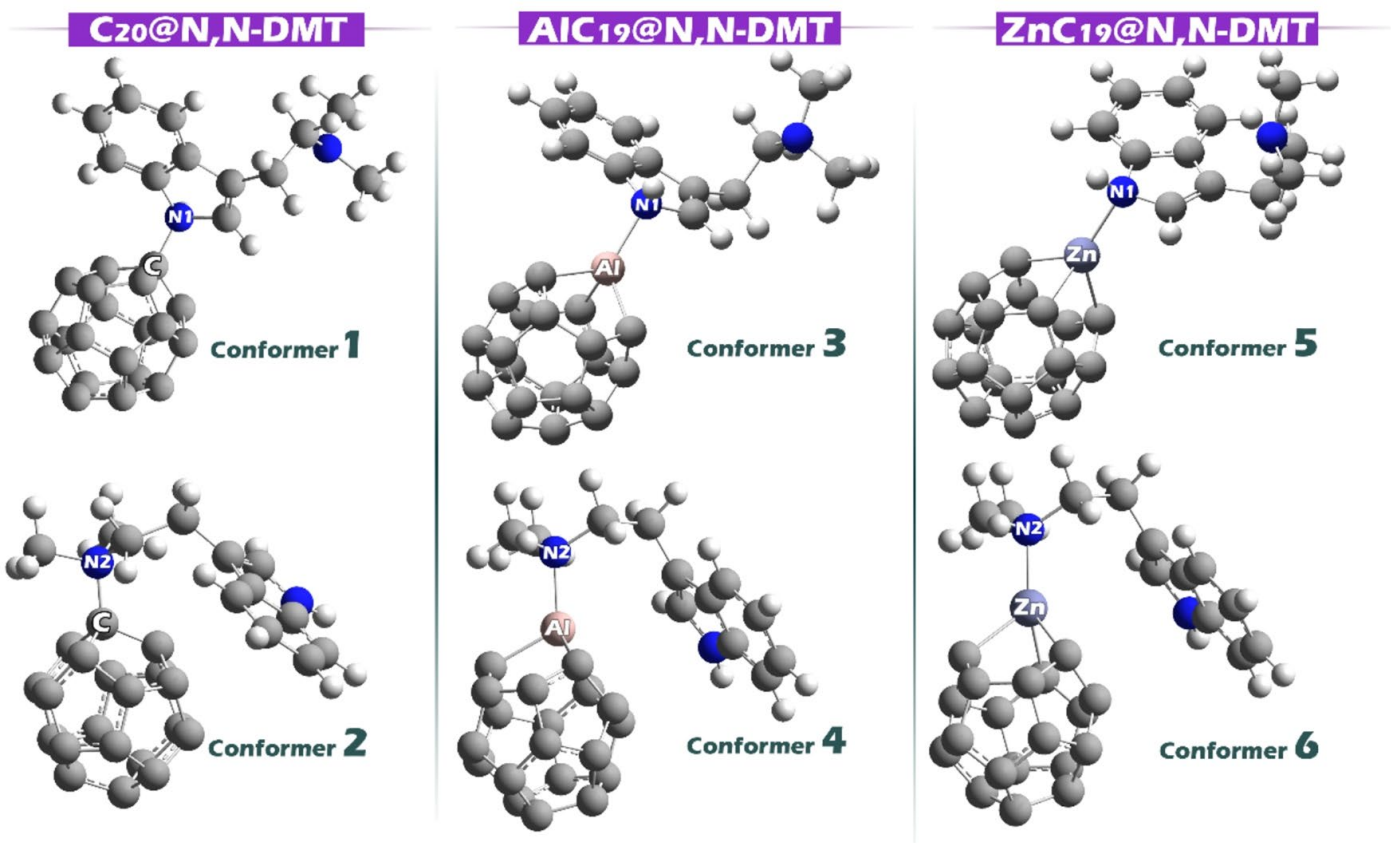
The optimized structure of each of the complexes designed in this paper.

In the MEP image, the regions with red and yellow colors on the N, N-DMT molecule indicate areas of high negative electrostatic potential, mainly around its nitrogen atoms and some aromatic portions. These are the likely electron-rich sites that can interact with positively charged or electron-deficient regions on other molecules. Looking at the C_20_, AlC_19_, and ZnC_19_ clusters, the blue-colored areas, especially near the Al and Zn atoms in AlC_19_ and ZnC_19_, represent positive electrostatic potential. These positive regions are the most probable sites for interaction, as they can attract the electron-rich (negative) parts of N, N-DMT. Therefore, the strongest interactions are expected between the negatively charged nitrogen and aromatic regions of N, N-DMT, and the positively charged dopant sites (Al and Zn) on the clusters. Based on the MEP findings, each of the desired complexes was designed. The optimal structure of each of these complexes is shown in Fig. 4.

#### Properties of HOMO/LUMO Frontier Orbitals

The electronic parameters energy gap (HLG), chemical softness (S), chemical hardness (η), chemical potential (µ), maximum charge transfer (ΔN_max_), and electrophilicity-based charge transfer (ECT) are critical in molecular design because they govern reactivity, stability, and charge-transfer behavior. The HOMO-LUMO gap (HLG) determines electronic excitation and conductivity, while chemical hardness (η) and softness (S) quantify molecular stability and polarizability, respectively. Chemical potential (µ) indicates electron donor/acceptor tendencies, ΔN_max_ predicts charge transfer capacity, and ECT evaluates the direction of charge transfer^50–52^. For this study, these properties were computationally analyzed for pristine C20 and its doped variants (AlC_19_, ZnC_19_) in the presence and absence of DMT, with detailed results presented in Table 2.

**Table 2:** Energy values ​​of orbitals HOMO (eV) and LUMO (eV) as well as HLG (eV), (eV), S (eV− 1), (eV), ∆Nmax and ECT for each of the conformers studied in this work.

ηµ									
Structure	Conformer	LUMO	HOMO	HLG	η	µ	S	∆*N*_max_	ECT
Sensor									
C_20_	**------**	-3.42	-5.37	1.95	0.97	-4.39	0.512	4.50	**------**
AlC_19_	**------**	-3.28	-6.18	2.92	1.46	-4.72	0.342	3.23	**------**
ZnC_19_	**------**	-3.06	-6.10	3.04	1.52	-4.58	0.328	3.01	**------**
Complex/Conformer									
C_20_@DMT	1	-2.26	-4.19	1.93	0.96	-3.22	0.518	3.34	-1.16
	2	-2.15	-4.09	1.94	0.97	-3.12	0.515	3.21	-1.29
AlC_19_@DMT	3	-2.43	-5.40	2.97	1.48	-3.91	0.336	2.63	-0.59
	4	-2.32	-5.29	2.97	1.48	-3.80	0.336	2.56	-0.67
ZnC_19_@DMT	5	-2.45	-5.47	3.02	1.51	-3.96	0.331	2.62	-0.39
	6	-2.51	-5.29	2.78	1.39	-3.90	0.359	2.80	-0.20

Based on the data presented in Table 2, a comparative analysis of the electronic properties before and after the interaction of each sensor with DMT reveals key changes that shed light on the sensing capability and electronic response of the designed structures.

Before interaction with DMT, the pristine sensors (C_20_, AlC_19_, and ZnC_19_) show distinct electronic characteristics. The C_20_ structure has the smallest HOMO-LUMO gap (HLG) of 1.95 eV, indicating higher electronic softness and potentially greater reactivity. In contrast, the doped structures, AlC_19_ and ZnC_19_, show significantly wider HLG values of 2.92 eV and 3.04 eV, respectively, suggesting increased chemical hardness (η) and greater electronic stability. These larger gaps are accompanied by lower softness (S) and lower maximum charge transfer capacity (∆N_max_), especially in the Zn-doped system. The chemical potential (µ) is also more negative in the doped structures, indicating a stronger tendency to retain electrons. These results demonstrate that doping increases the electronic stability of the sensor molecules and reduces their intrinsic reactivity, as already observed in the cohesive energy and MEP analyses.

Upon interaction with DMT, noticeable shifts in all electronic descriptors occur across the sensors. For the C_20_@DMT complex (conformers 1 and 2), the HLG slightly decreases to around 1.93–1.94 eV, which is close to the original value for C_20_, indicating a relatively minor electronic rearrangement. The chemical potential increases significantly (from − 4.39 eV to -3.12/-3.22 eV), and ∆Nmax decreases (from 4.50 to around 3.2–3.3), suggesting a clear charge redistribution and moderate charge transfer upon complex formation. Importantly, the ECT (electron charge transfer) values of -1.16 and − 1.29 eV reflect a substantial flow of electrons from DMT to C_20_, consistent with the electrostatic complementarity shown in the MEP maps.

For AlC_19_@DMT (conformers 3 and 4), the HLG slightly increases to 2.97 eV, similar to the original AlC_19_ sensor. However, the chemical potential increases (becomes less negative) from − 4.72 to -3.91/-3.80 eV, and ∆Nmax drops to around 2.6, indicating a stronger interaction with DMT and a greater impact on electron distribution. The ECT values of -0.59 and − 0.67 eV suggest moderate electron transfer, pointing to an effective but less pronounced interaction than that observed for C_20_.

In the case of ZnC_19_@DMT (conformers 5 and 6), the HLG remains close to the original ZnC_19_ values (2.78–3.02 eV), with a minor decrease. However, the chemical potential increases from − 4.58 to about − 3.90 eV, and softness slightly improves. Notably, the ECT values of -0.39 and − 0.20 eV are smaller than those in the C_20_ and AlC_19_ complexes, indicating a weaker electron transfer process despite the strong electrostatic complementarity observed in the MEP contours.

**Fig. 5:**
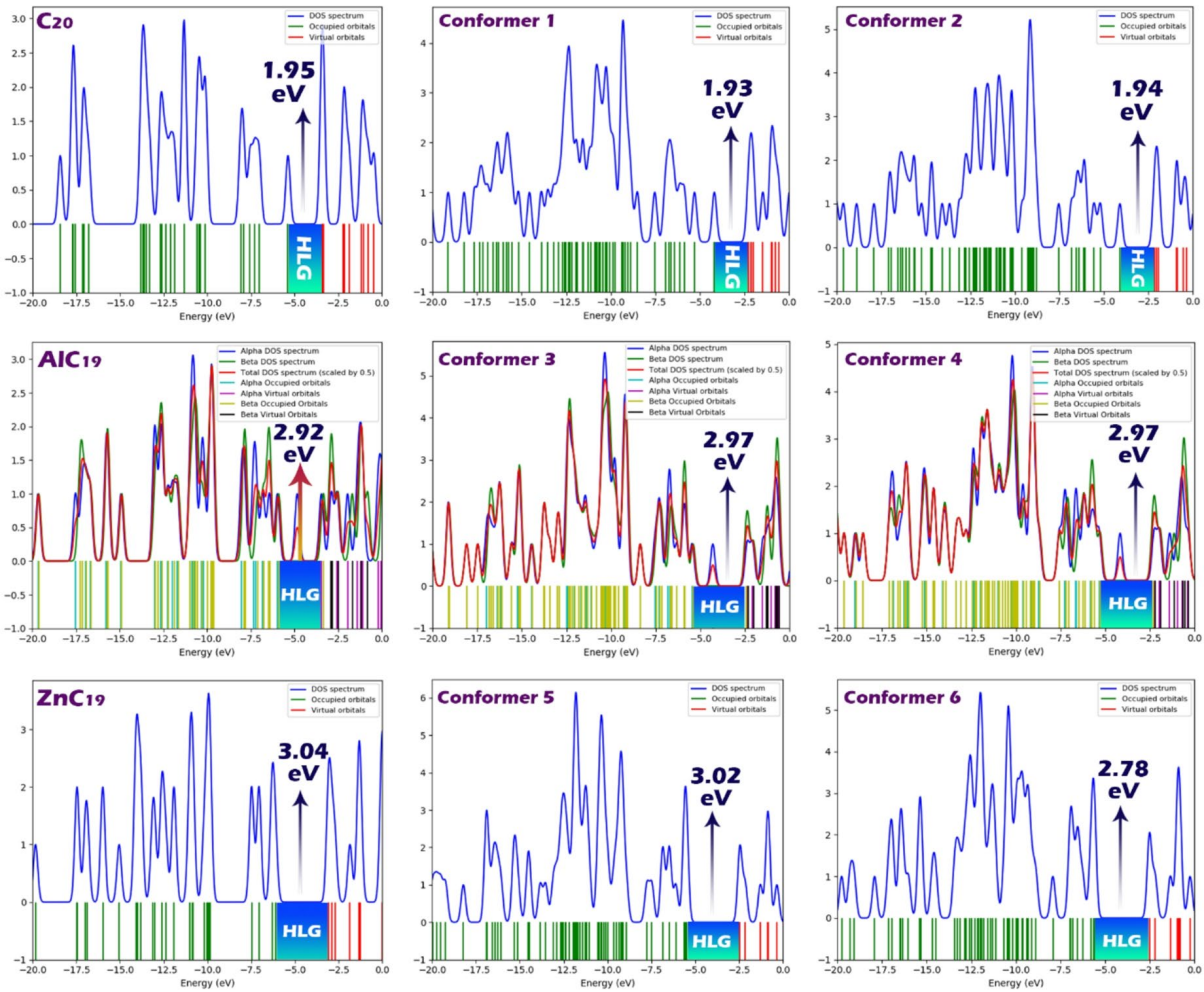
DOS plots for each of the designed sensors in the presence and absence of N, N-DMT.

Density of States (DOS) contours provide a graphical representation of the distribution of electronic states at various energy levels within a molecule. They serve as a powerful visual tool to understand the electronic structure, particularly the energy gap between the HOMO and the LUMO. The energy gap is visually identified in DOS plots as the region with no electronic states between the occupied and unoccupied levels (Fig. 5)^53,54^. In this study, DOS contours were generated for each of the molecular structures investigated, including both the sensors and their complexes with DMT. These contours allow for a direct comparison of how doping and molecular interaction influence the electronic structure and energy gap of the systems. The DOS diagrams clearly confirm the trends reported in Table 2 for the HOMO-LUMO gap (HLG). For example, the doped structures (AlC_19_ and ZnC_19_) show wider gaps in their DOS plots, in agreement with their higher HLG values, indicating enhanced electronic stability. These visual confirmations further corroborate the electronic analysis (Reported in Table 2).

**Fig. 6:**
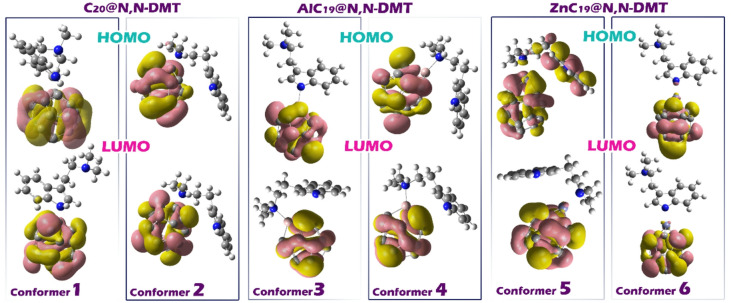
Distribution of HOMO/LUMO frontier orbitals in each of the designed complexes.

Examining the spatial distribution of the HOMO and LUMO is essential for understanding the reactivity, charge transfer capability, and sensing behavior of molecular systems. These frontier orbitals play a key role in chemical interactions, as the HOMO typically donates electrons while the LUMO accepts them. Their spatial localization reveals which parts of the molecule are most involved in electronic transitions or interactions with other species (see Fig. 6)^55,56^. In the studied complexes, analysis of the HOMO and LUMO distributions shows that both orbitals are entirely localized on the sensor molecules, not on the DMT analyte. This is a favorable characteristic in sensor design, as it indicates that the sensor is primarily responsible for the electronic response, rather than the analyte altering the orbitals directly. Such localization ensures that any changes in electronic properties (such as shifts in energy levels or changes in conductivity) are due to interaction with the analyte and not due to fundamental changes in the sensor’s orbital structure. This feature enhances sensor reliability and selectivity, as the sensor maintains its intrinsic electronic framework while still responding to external molecules through measurable changes.

#### Adsorption energy, recovery time, and electrical conductivity

To investigate the performance of a molecule as a sensor, three key parameters are commonly evaluated: adsorption energy (Eads), recovery time (τ), and electrical conductivity (σ)^57^. These properties provide a comprehensive understanding of how effectively a sensor can detect and respond to target analytes. Adsorption energy indicates the strength of the interaction between the sensor and the analyte. A more negative adsorption energy reflects a stronger and more stable binding, which is essential for effective detection^58^. Recovery time reflects how quickly the sensor can return to its original state after interaction, which is critical for reusability and real-time sensing applications^59^. Electrical conductivity, often inferred from the HOMO-LUMO gap, determines how readily the sensor can translate molecular interactions into measurable electrical signals^60^. Each of these parameters was studied computationally for the pristine C_20_ molecule and its doped forms (AlC_19_ and ZnC_19_), in the absence/presence of N, N-DMT, and the results are reported in Table 3.

**Table 3:** Adsorption energies (Eads) of Various Conformers of C20, AlC19, and ZnC19 Complexes with N, N-DMT analyte. The most stable conformer for each complex is highlighted based on the most negative adsorption energy.

Structure	Conformer	E_ads_ (kcal.mol^− 1^)
C_20_@ N, N-DMT	1	-0.62
	2	-20.08
AlC_19_@ N, N-DMT	3	-33.25
	4	-49.57
ZnC_19_@ N, N-DMT	5	-22.59
	6	-8.78

To analyze the data reported in Table 3, we first determine the most stable conformer for each complex based on the given adsorption energy (Eads) values. A more negative value for Eads indicates a stronger interaction and therefore a more stable conformer. For the C_20_@N, N-DMT complex, conformer 2 is clearly the more stable, as it has a more negative Eads value. Similarly, for the AlC_19_@N, N-DMT complex, conformer 4 is the most stable due to its more negative adsorption energy. For the ZnC_19_@N, N-DMT complex, conformer 5 is the most stable conformer because its Eads value is more negative. After identifying the most stable conformers, further calculations were performed using these conformers. This is because the most stable form of a complex is the one most likely to exist in reality, and focusing on it ensures that calculations reflect the most desirable and physically realistic configuration of the system.

Comparing the most stable conformers across the three complexes, we see that the AlC_19_@ N, N-DMT complex (Conformer 4) has the most negative Eads value of -49.57 kcal/mol, indicating that it is the most stable of all the complexes. In comparison, the ZnC_19_@ N, N-DMT complex (Conformer 5) is slightly less stable with an Eads of -22.59 kcal/mol, while the C_20_@ N, N-DMT complex (Conformer 2) has the least stable form with an Eads of -20.08 kcal/mol. Thus, the AlC_19_@ N, N-DMT complex exhibits the strongest interaction, followed by ZnC_19_@ N, N-DMT and C_20_@ N, N-DMT, respectively.

In summary, the results suggest that AlC_19_ is the most suitable material for DMT absorption and removal applications due to its strong binding affinity, as indicated by its highly negative adsorption energy. This strong interaction makes it effective at trapping and removing DMT from systems. However, for sensing applications, further investigation is needed into additional parameters, such as recovery time, electrical conductivity, and maximum absorption wavelength. Each of these parameters is examined in detail below.

**Table 4:** Recovery time () and electrical conductivity () values for the most stable conformers of C20, AlC19, and ZnC19, with and without N, N-DMT adsorption.

τσ			
Structure	$\:\boldsymbol{\tau\:}$ (s)	Log10 (τ)	($\:\boldsymbol{\sigma\:}$) (S.m^− 1^)
C_20_	**-----**	**-----**	2.08 × 10^9^
AlC_19_	**-----**	**-----**	1.71 × 10^9^
ZnC_19_	**-----**	**-----**	2.67 × 10^9^
C_20_@N, N-DMT (Conformer 2)	5.34 × 10^2^	2.73	2.08 × 10^9^
AlC_19_@ N, N-DMT (Conformer 4)	2.28 × 10^24^	2.36	1.69 × 10^9^
ZnC_19_@ N, N-DMT (Conformer 5)	3.70 × 10^4^	4.57	1.67 × 10^9^

A careful interpretation and comparison of the recovery time (τ) and electrical conductivity (σ) values ​​for the most stable conformers of the three complexes allows us to conclude on the suitability of C_20_, AlC_19_ and ZnC_19_ for N, N-DMT adsorption/removal or sensing applications (Table 4).

**Fig. 7:**
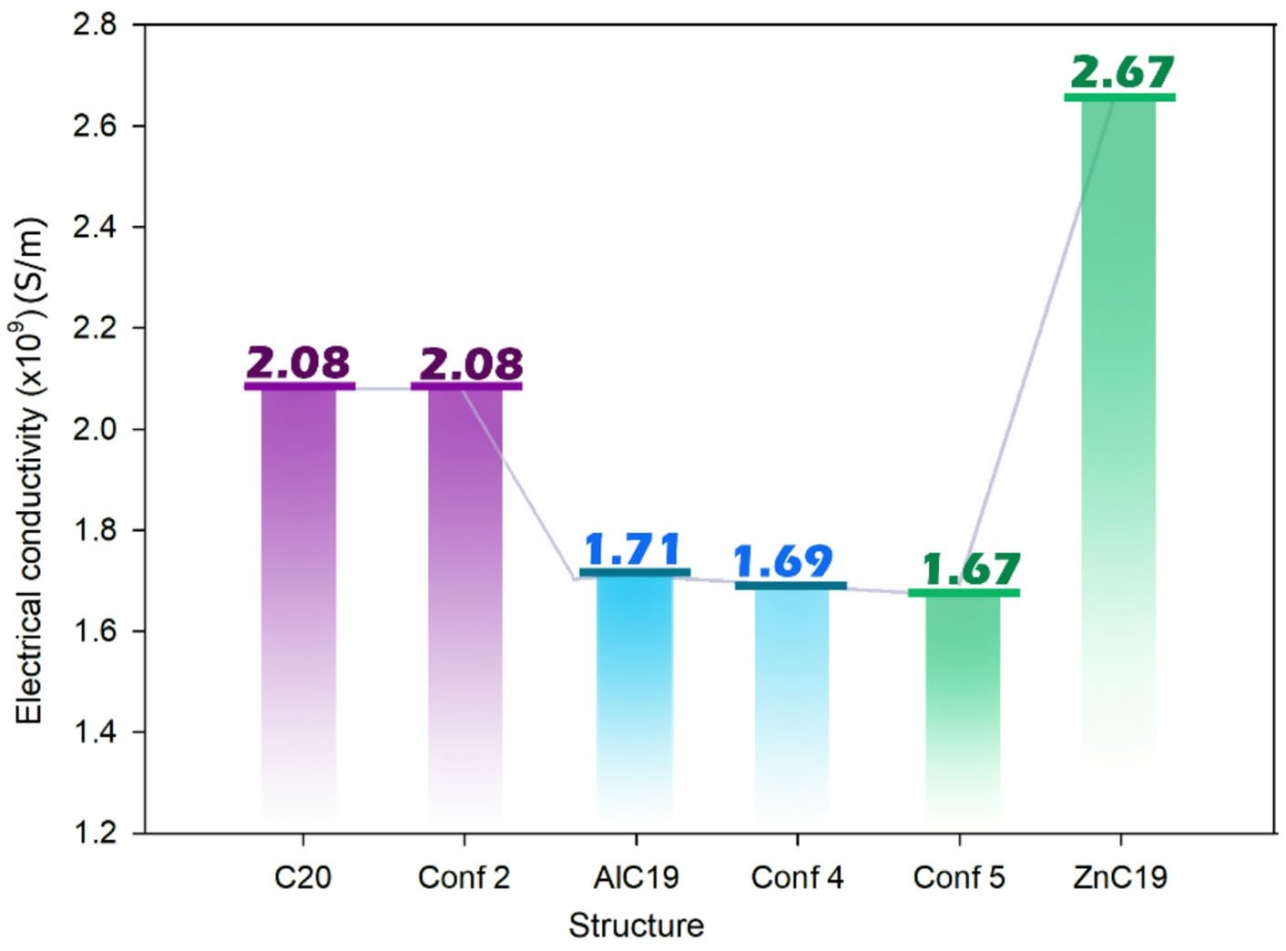
The trend of electrical conductivity changes in the C_20_, AlC_19_, and ZnC_19_ sensors and the most stable form of their complex with N, N-DMT.

In this study, the electrical conductivity values presented are derived from theoretical calculations based on the HOMO-LUMO energy gap, which provides a qualitative estimate of the conductivity behavior in the material upon interaction with N, N-DMT. These values are indicative of the electronic changes within the system and suggest trends in conductivity, but they do not represent direct physical measurements of conductivity (σ). Therefore, it is important to note that the conductivity values ​​reported in this study should be considered as qualitative rather than quantitative estimates. In the absence of N, N‑DMT (that is, the analyte), the conductivities are: C_20_≈2.08 × 10^9^ S/m, AlC_19_ ≈ 1.71 × 10^9^ S/m, and ZnC_19_ ≈ 2.67 × 10^9^ S/m. Upon adsorption of N, N‑DMT (in the more stable conformers) the conductivities become: C_20_@N, N‑DMT ≈ 2.08 × 10^9^ S/m, AlC_19_@N, N‑DMT ≈ 1.69 × 10^9^ S/m, and ZnC_19_@N, N‑DMT ≈ 1.67 × 10^9^ S/m. Thus, adsorption of N, N‑DMT produces only marginal changes in conductivity for C_20_ and AlC_19_ (from 2.08→2.08 × 10^9^ and 1.71→1.69 × 10^9^ respectively), whereas for ZnC_19_ the conductivity drops from 2.67 × 10^9^ to 1.67 × 10^9^ S/m (a significant change) (See Fig. 7). This notable drop suggests that ZnC_19_ is more responsive (in electrical-terms) to adsorption of the analyte molecule, which is advantageous for sensing: the target molecule’s presence modulates the conductivity significantly. In contrast, the very small conductivity change seen for AlC_19_ (and C_20_) indicates that after adsorption the electrical properties remain essentially unchanged.

The observed changes in electrical conductivity upon adsorption of N, N-DMT are directly relevant to electrochemical sensing mechanisms. In practical electrochemical sensors, conductivity variations alter the charge transfer resistance and electron transport properties of the sensing material. When the ZnC_19_ sensor adsorbs N, N-DMT, a significant decrease in conductivity (from 2.67 × 10^9^ to 1.67 × 10^9^ S/m) indicates a reduction in electron mobility due to electron transfer from the analyte to the sensor, as supported by the negative ECT values. This change can be measured experimentally as a shift in current or impedance in electrochemical setups such as cyclic voltammetry (CV) or electrochemical impedance spectroscopy (EIS). The larger conductivity change in ZnC_19_ compared to AlC_19_ and pristine C_20_ suggests a more pronounced electrical response, making it a promising candidate for amperometry or conductometric detection of N, N-DMT in real-time sensing applications.

**Fig. 8:**
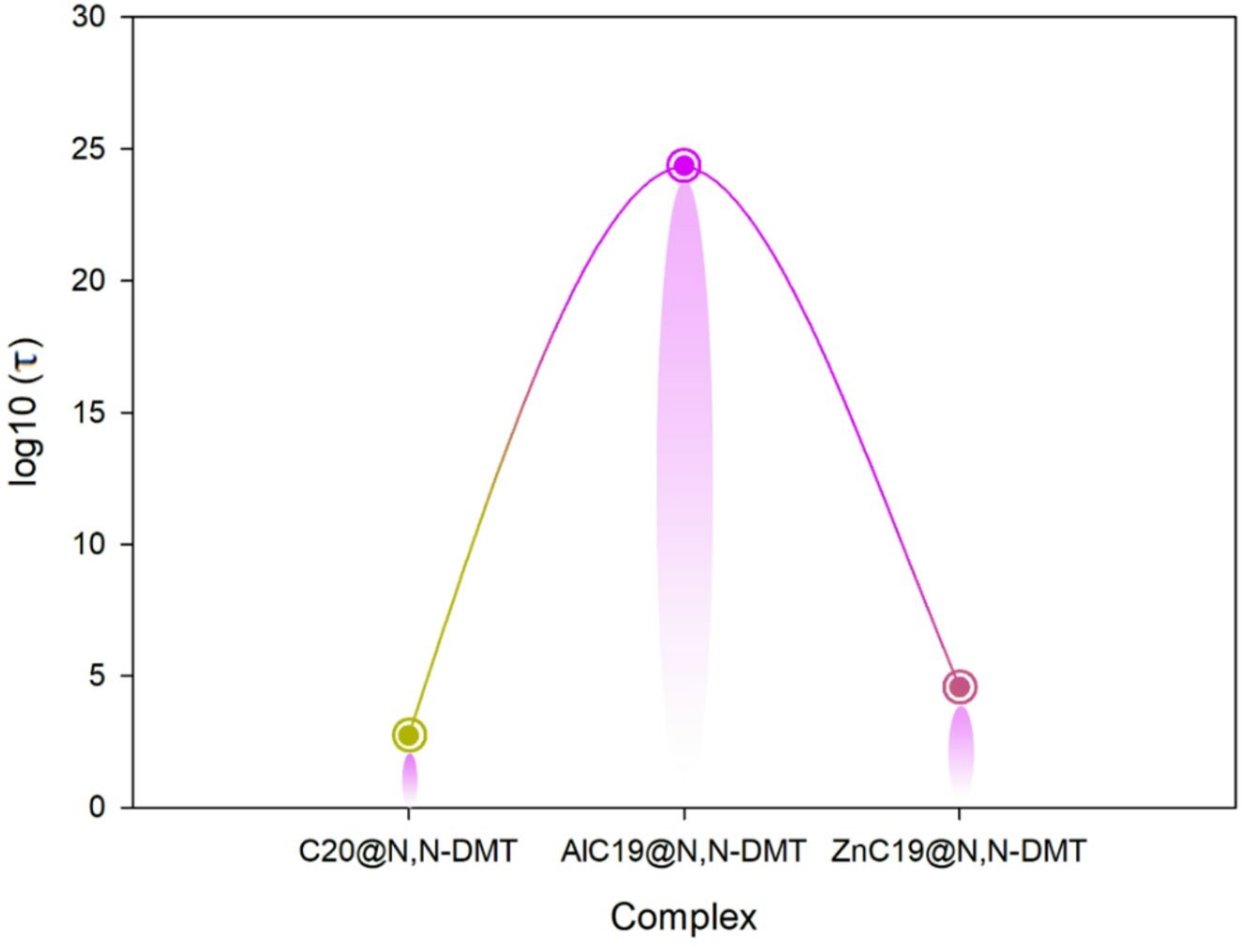
Logarithmic (log10) comparison of recovery time (τ) for C_20_-, AlC_19_-, and ZnC_19_-based systems upon N, N-DMT adsorption, illustrating relative desorption trends derived from computational analysis.

In the next step, the recovery time was studied. For C_20_@N, N‑DMT the recovery time is ~ 5.34 × 10^2^ s. For AlC_19_@N, N‑DMT the recovery time is enormous: ~2.28 × 10^24^ s (on the order of 10^24^ s, effectively irreversible under ambient conditions). For ZnC_19_@N, N‑DMT the recovery time is ~ 3.70 × 10^24^ s. A logarithmic scale was used to highlight relative differences in recovery behavior rather than absolute quantitative values, and the results are reported in Table 8. According to the results obtained, the recovery behavior of the studied systems exhibits pronounced relative differences upon N, N-DMT adsorption. AlC_19_@N, N-DMT displays a substantially longer recovery trend compared to C_20_@N, N-DMT and ZnC_19_@N, N-DMT, suggesting stronger analyte-surface interaction. In contrast, the comparatively lower recovery trends observed for C_20_- and ZnC_19_-based systems indicate faster desorption characteristics (Fig. 8). It is emphasized that these observations are qualitative and derived from computational analysis.

In general, in sensing applications, one desires short recovery times so that the sensor can be reused quickly; in adsorption/removal applications one often desires long retention (long recovery time) to hold on to the target molecule rather than releasing it. Accordingly, the extremely long recovery time of AlC_19_@N, N‑DMT suggests very strong binding/very slow desorption (suitable for removal/capture, less suitable for rapid sensing). ZnC_19_ has a moderately long recovery time (10^4^-10^5^ s) which is longer than would be ideal for high-throughput real‑time sensing but may be acceptable if the sensor is used in slower protocols or single‐use cycles, especially given its good conductivity response.

Ultimately, these results suggest that AlC_19_ is impractical for reusable sensing and only useful for permanent adsorption/adsorption (not as a sensor), because it binds strongly to N, N-DMT, is hardly electronically altered, and retains the target molecule essentially irreversibly (which is useful in adsorption applications). On the other hand, ZnC_19_ can be targeted for sensing applications: its conductivity is significantly modulated by the presence of the analyte (making it sensitive), and although its recovery time is slower than ideal, it remains within an acceptable range for certain sensing contexts, thus demonstrating acceptable accuracy and reusability.

#### UV spectrum

**Fig. 9:**
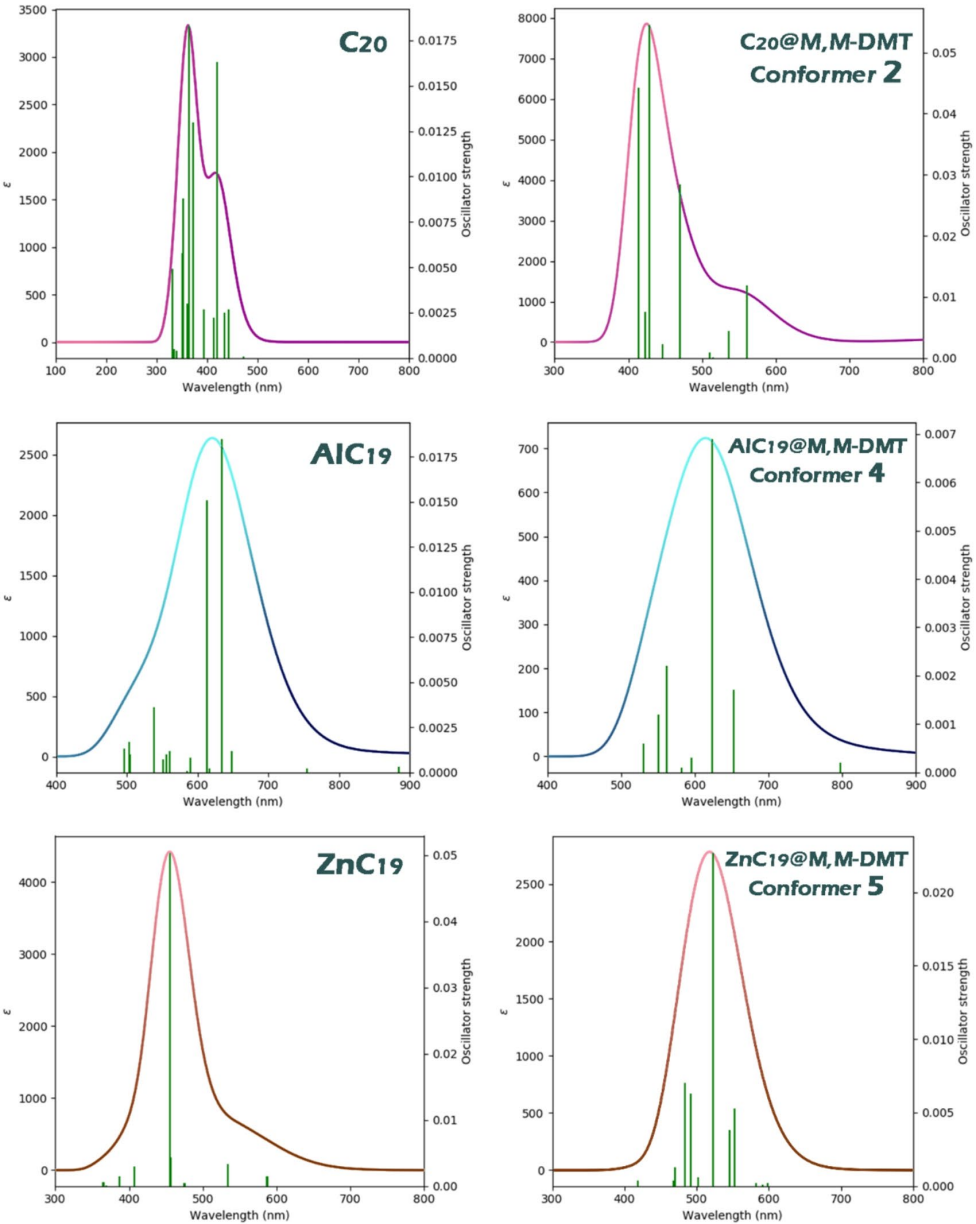
Colorimetric response of C_20_, AlC_19_, and ZnC_19_ upon interaction with N, N-DMT, showing the shift in λmax.

Examining the maximum adsorption wavelength and exciton energy is essential for evaluating a structure’s potential as a colorimetric sensor because these optical parameters directly determine how the material’s color or light-absorption behavior changes when an analyte is present. A shift in the maximum absorption wavelength indicates that interaction with the analyte alters the electronic transitions of the sensor, producing a measurable color change. Exciton energy provides insight into how strongly the analyte affects the sensor’s excited-state properties, with significant variations signaling an observable optical response. Together, these quantities reveal whether the sensor can produce clear, detectable colorimetric signals upon analyte binding^61,62^. Each of these values ​​was calculated, and the results were reported in Table 5. Additionally, the UV spectra of each structure, both in the presence/absence of N, N-DMT, are visually shown in Fig. 9.

**Table 5:** Maximum absorption wavelength (max), oscillator strengths (ƒ), Transition contributions of HOMO to LUMO, and excitation wavelength (Eex) values for C20, AlC19, and ZnC19 in the presence and absence of N, N-DMT, highlighting the colorimetric response of each complex.

λ				
Structure	λmax	Eex	ƒ	Transition contributions of HOMO to LUMO
C_20_	363	3.41	0.0183	HOMO_4_→LUMO (31%)
AlC_19_	634	1.95	0.0185	HOMO_(B)_→LUMO_(B)_ (38%)
ZnC_19_	455	2.72	0.0504	HOMO→L + 2 (68%)
C_20_@N, N-DMT (Conformer 2)	428	2.89	0.0546	HOMO_2_→LUMO (77%)
AlC_19_@ N, N-DMT (Conformer 4)	623	1.98	0.0069	HOMO_(B)_→LUMO_(B)_ (60%)
ZnC_19_@ N, N-DMT (Conformer 5)	523	2.37	0.0227	HOMO_2_→LUMO (49%)

When comparing the data for the sensors C_20_, AlC_19_, and ZnC_19_ in the presence and absence of N, N-DMT, we are looking at two important parameters: the maximum absorption wavelength (λ_max_) and the excitation wavelength (E_ex_). These parameters are critical for understanding how each material interacts with light, which is key to its performance in colorimetric sensing applications.

For C_20_, in the absence of N, N-DMT, the λ_max_ is 363 nm, and the E_ex_ is 3.41, indicating that C_20_ absorbs light at a relatively short wavelength and requires higher energy to be excited. When C_20_ interacts with N, N-DMT, the λ_max_ shifts to 428 nm, which is a noticeable shift toward a longer wavelength, suggesting a change in the electronic structure of C_20_ upon binding with N, N-DMT. The E_ex_ decreases slightly to 2.89, indicating a slight change in the excitation energy required.

For AlC_19_, the λ_max_ in the absence of N, N-DMT is much higher, at 634 nm, and the E_ex_ is 1.95. This indicates that AlC_19_ absorbs light in the red region, requiring less excitation energy compared to C_20_. In the presence of N, N-DMT, the λ_max_ shifts to 623 nm, which is a smaller shift compared to C_20_, indicating that the interaction with N, N-DMT has less of an effect on its absorption properties. The E_ex_ slightly increases to 1.98, showing minimal change in the excitation energy required.

For ZnC_19_, the λ_max_ in the absence of N, N-DMT is 455 nm, with an E_ex_ of 2.72. In the presence of N, N-DMT, the λ_max_ shifts to 523 nm, a significant shift toward a longer wavelength, indicating a notable change in the material’s interaction with light upon binding with N, N-DMT. The E_ex_ decreases to 2.37, indicating a slight reduction in the excitation energy required.

The data from Table 5 indicates the oscillator strengths (ƒ) and transition contributions of the HOMO to LUMO for C_20_, AlC_19_, and ZnC_19_, both with and without N, N-DMT. The oscillator strength values are higher for ZnC_19_ (0.0504) compared to C_20_ (0.0183) and AlC_19_ (0.0185), suggesting a stronger transition and potentially better colorimetric response for ZnC_19_. Upon adsorption of N, N-DMT, C_20_ shows a significant increase in oscillator strength (0.0546), indicating a stronger response. In contrast, AlC_19_@N, N-DMT shows a lower oscillator strength (0.0069), suggesting a weaker response upon interaction. Transition contributions show that the HOMO to LUMO transition is dominant for all structures, with C_20_ and ZnC_19_ showing the largest contributions to this transition in the presence of N, N-DMT. Overall, ZnC_19_ exhibits the most significant potential for colorimetric response, while AlC_19_ shows the weakest.

When analyzing these shifts, we can see that ZnC_19_ exhibits the most significant change in λ_max_ (from 455 nm to 523 nm), indicating a pronounced interaction with N, N-DMT. This large shift in λ_max_ suggests that ZnC_19_ undergoes a noticeable color change when it binds with N, N-DMT. This change in color can be expected to be visible to the naked eye, especially as the λ_max_ moves from the blue region (455 nm) to the green region (523 nm) of the visible spectrum. Such a shift would produce a visible color change that can be easily detected without the need for sophisticated instrumentation, making ZnC_19_ a strong candidate for a colorimetric sensor.

Although C_20_ can also function as a colorimetric sensor, the colorimetric response of ZnC_19_ is notably stronger. The key factor here lies in the extent of the shift in the maximum absorption wavelength (λ_max_) upon interaction with N, N-DMT. For C_20_, the λ_max_ shifts from 363 nm (in the absence of N, N-DMT) to 428 nm (in the presence of N, N-DMT). This shift indicates a change in the material’s absorption properties, but it remains within the ultraviolet/visible spectrum and is relatively modest. While this shift is detectable, it falls towards the edge of the visible range, which may not result in a highly noticeable or dramatic color change. The colorimetric response of C_20_ is thus weaker compared to ZnC_19_ because the shift in absorption does not produce a strong, visible color change that is easily perceptible to the naked eye.

In contrast, ZnC_19_ exhibits a much more significant change in λ_max_, shifting from 455 nm (in the absence of N, N-DMT) to 523 nm (in the presence of N, N-DMT). This represents a substantial shift from the blue region to the green region of the visible spectrum. Such a change is far more pronounced and results in a much more noticeable color change, making ZnC_19_’s colorimetric response much stronger and easier to detect visually. The visible color shift from blue to green in ZnC_19_ is more dramatic and can be observed without specialized equipment, making it a more effective colorimetric sensor.

The predicted shift in the Δλ_max_ for ZnC_19_ upon interaction with N, N-DMT (from 455 nm to 523 nm) corresponds to a visible color change from blue to green. Such a shift (~ 68 nm) is well within the detectable range of standard UV-Vis spectrophotometers and is likely observable even with the naked eye under appropriate conditions. In practical sensing applications, this colorimetric response could be measured using portable spectrophotometers or even smartphone-based colorimetric assays, enabling on-site detection. The significant oscillator strength (ƒ) further supports the feasibility of experimental detection, as it indicates a strong electronic transition conducive to measurable absorbance changes. Future experimental validation should focus on synthesizing ZnC_19_-based sensors and testing their optical response in solution or thin-film formats to confirm the predicted shifts and assess real-world applicability.

Therefore, while C_20_ can indeed act as a colorimetric sensor, the color change it undergoes upon interacting with N, N-DMT is less striking compared to ZnC_19_, which exhibits a stronger and more visually detectable colorimetric response. This makes ZnC_19_ the superior choice when a more pronounced and easily visible color change is required for sensing applications. To better understand changes in the maximum absorption wavelength, the UV spectra of the proposed sensors in the presence and absence of N, N-DMT are shown in Fig. 9.

#### NBO analysis

NBO analysis provides insights into bonding and non-bonding interactions by evaluating donor-acceptor orbital interactions within the molecular system. Among the various electronic transitions identified in this analysis, the most important include σ → σ*, π → π*, lone pair (LP) → σ*, and LP → π* interactions, with π → π* transitions being dominant. In this study, we use NBO analysis to examine how the complexes C_20_, AlC_19_, and ZnC_19_ interact with N, N-DMT, focusing on the second-order perturbation energy E^(2)^ values that quantify the strength of these interactions. The E^(2)^ value is a measure of the stabilization energy resulting from the interaction between a donor orbital (such as a lone pair (LP), a σ bond, or a π bond) and an acceptor orbital (such as an anti-bonding σ* or π* orbital). These interactions are a fundamental part of molecular bonding, and the magnitude of E^(2)^ indicates the strength of orbital interaction. E^(2)^ is calculated as the energy change that occurs when electron density is transferred from the donor orbital to the acceptor orbital. The larger the E^(2)^ value, the greater the extent of this transfer and the stronger the interaction between the donor and acceptor orbitals^63,64^.

**Table 6:** Calculated values for the second-order perturbation energy matrix (E2) using NBO analysis in each of the conformers studied in this work.

Complex	Donor (i)	Type	Acceptor (j)	Type	E^(2)^ kcal.mol^− 1^	E(j)-E(i) a.u.	F(i, j) a.u.
C_20_@DMT (Conformer 2)	C_1_-C_2_	$\:\sigma\:$	C_1_-C_12_	$\:{\sigma\:}^{*}$	3.24	1.23	0.056
	C_4_-C_8_	$\:\pi\:$	C_7_-C_11_	$\:{\pi\:}^{*}$	1.30	0.67	0.029
	N_32_	LP (1)	C_30_-C_31_	$\:{\pi\:}^{*}$	5.33	0.36	0.039
AlC_19_@DMT (Conformer 4)	C_2_-C_5_	$\:\sigma\:$	C_4_-C_8_	$\:{\sigma\:}^{*}$	2.06	1.07	0.059
	C_24_-C_25_	$\:\pi\:$	C_22_-C_23_	$\:{\pi\:}^{*}$	10.30	0.29	0.071
	C_11_	LP (1)	C_7_-C_8_	$\:{\pi\:}^{*}$	30.99	0.15	0.099
ZnC_19_@DMT (Conformer 5)	C_20_-C_21_	$\:\sigma\:$	C_22_-N_32_	$\:{\sigma\:}^{*}$	5.98	1.16	0.074
	C_9_-C_10_	$\:\pi\:$	C_1_-C_12_	$\:{\pi\:}^{*}$	25.66	0.33	0.083
	C_4_	LP (1)	C_1_-C_3_	$\:{\pi\:}^{*}$	41.85	0.14	0.082

For C_20_@DMT, the (E^2^) values suggest that while the complex forms a stable interaction with N, N-DMT, its sensor capabilities are somewhat weaker compared to AlC_19_@DMT and ZnC_19_@DMT. The (E^2^) value of 3.24 kcal/mol for this interaction indicates a moderate electron donation from the σ bond of C_1_-C_2_ to the anti-bonding σ orbital of C_1_-C_12_. The energy difference (E(j)-E(i) = 1.23 a.u.) and interaction strength (F(i, j) = 0.056) suggest a moderate overlap between the donor and acceptor orbitals, contributing to the overall stability of the complex but not as strongly as the interactions in AlC_19_@DMT or ZnC_19_@DMT. This indicates that C_20_@DMT binds to N, N-DMT, but the interaction is less significant, leading to weaker sensor capabilities. The π → π interaction in C_20_@DMT is even weaker, with an (E²) value of 1.30 kcal/mol. The larger energy difference (E(j)-E(i) = 0.67 a.u.) and the low interaction strength (F(i, j) = 0.029) confirm that this interaction plays a minimal role in stabilizing the complex. This weak interaction suggests that C_20_@DMT has a lower sensitivity compared to the other complexes, as π-electron interactions are less effective in enhancing binding with N, N-DMT. The LP → π interaction in C_20_@DMT is stronger than the π-π* interaction but still moderate compared to AlC_19_@DMT and ZnC_19_@DMT, with an (E^2^) value of 5.33 kcal/mol. The energy difference (E(j)-E(i) = 0.36 a.u.) suggests good overlap between the donor and acceptor orbitals, while the interaction strength (F(i, j) = 0.039) indicates that this lone pair donation contributes to stabilizing the complex, though to a lesser extent than in the other complexes. This interaction ensures that C_20_ has some binding affinity with N, N-DMT, but the overall strength of the complex is weaker compared to AlC_19_@DMT and ZnC_19_@DMT. These interactions suggest that C_20_@DMT would be effective in sensing applications but with lower sensitivity and binding strength compared to AlC_19_@DMT and ZnC_19_@DMT. The complex’s sensor capabilities would likely be more suited for applications where moderate binding affinity is sufficient, and where a less responsive interaction is acceptable. While C_20_@DMT would be able to detect N, N-DMT, it would likely exhibit slower responses and weaker signals compared to the other complexes due to the relatively weaker interactions (Table 6).

For AlC_19_@DMT, the most significant interaction is the LP → π* interaction, with a high (E^2^) value of 30.99 kcal/mol, which is the largest among the interactions in this complex. This interaction indicates strong electron donation from the lone pair on C_11_ to the π* orbital of C_7_-C_8_, resulting in a highly stable complex. The small energy difference (0.15 a.u.) and the strong interaction strength (F(i, j) = 0.099) confirm that this interaction is the dominant contributor to the stability of AlC_19_@DMT. The π → π* interaction ((E²) = 10.30 kcal/mol) further strengthens the complex, contributing to the overall binding strength. The *σ → σ interaction (E2 = 2.06 kcal/mol)**, while present, plays a lesser role in stabilizing the complex compared to the lone pair and π-electron interactions. These strong interactions suggest that AlC_19_@DMT would be effective in adsorption-based sensing applications, where the sensor’s ability to absorb and bind to N, N-DMT is crucial. The strong LP → π* interaction ensures that AlC_19_@DMT can form a stable complex with the analyte, making it highly responsive to the presence of N, N-DMT. While AlC_19_ forms a very stable complex with N, N-DMT, it is more suited to applications requiring strong binding and adsorption rather than rapid reusability. The strong binding may limit its recovery time, making it ideal for applications where long-term trapping or removal of the analyte is desired.

ZnC_19_@DMT exhibits even stronger interactions, particularly due to the LP → π* interaction, which has the highest (E²) value of 41.85 kcal/mol in this study. This high value indicates an extremely strong electron donation from the lone pair on C_4_ to the π* orbital of C_1_-C_3_ in N, N-DMT, leading to a very stable complex. The small energy difference (E(j)-E(i) = 0.14 a.u.) and strong interaction strength (F(i, j) = 0.082) suggest a highly favorable orbital overlap, making the complex very stable. In addition, the π → π interaction ((E²) = 25.66 kcal/mol)* between C_9_-C_10_ and C_1_-C_12_ further strengthens the complex, indicating that ZnC19 has a strong π-electron interaction with N, N-DMT. The σ → σ interaction ((E²) = 5.98 kcal/mol)* also contributes to the overall stability, although it is less significant than the lone pair and π-electron interactions. The strong interactions in ZnC_19_@DMT suggest that ZnC_19_ will be highly effective in electrochemical sensing applications. The significant charge redistribution upon binding with N, N-DMT will lead to pronounced changes in the material’s electrical conductivity, making it highly sensitive to the presence of N, N-DMT. The strong binding affinity, particularly through the LP → π* interaction, ensures that ZnC_19_@DMT forms a stable complex with N, N-DMT, leading to a measurable electrical response. The relatively moderate recovery time (~ 3.70 × 10^4^ s) suggests that while ZnC_19_ binds strongly to N, N-DMT, the sensor can still be reset and reused over a reasonable time frame, making it suitable for high-sensitivity, real-time detection applications.

In both AlC_19_@DMT and ZnC_19_@DMT, the LP → π* interaction promotes electron transfer from the sensor to the analyte. This process causes changes in the electron density and orbital overlap, leading to measurable shifts in the sensor’s properties. For electrical sensors, this translates into changes in conductivity as the electron density within the sensor material shifts. For colorimetric sensors, the electron transfer can alter the absorption spectra of the complex, leading to visible color changes. These shifts in electronic properties provide the signal that is detected and used for sensing applications.

#### Practical implications of irreversible vs. reversible adsorption for sensing

The marked differences in recovery time between AlC_19_ and ZnC_19_ highlight a fundamental trade-off in sensor design between binding affinity and reusability. AlC_19_ exhibits effectively irreversible adsorption (τ ≈ 2.28 × 10^24^ s), which is advantageous for applications requiring permanent capture or removal of N, N-DMT, such as in filtration systems, environmental remediation, or forensic sample preservation. In such contexts, strong, irreversible binding ensures long-term retention and prevents analyte release. However, for real-time sensing applications, irreversible adsorption is detrimental because it precludes sensor regeneration, limiting the device to single-use applications and hindering continuous monitoring.

In contrast, ZnC_19_ shows a more practical recovery time (~ 3.70 × 10^4^ s, ~ 10 h), indicating reversible adsorption under ambient conditions. This reversibility is crucial for reusable sensors, allowing periodic refreshment of the active surface and enabling repeated measurements. Although its recovery is slower than ideal for rapid, high-throughput sensing, it remains within an acceptable range for many field-deployable or laboratory-based sensing protocols, especially when paired with thermal or chemical regeneration strategies. The reversible nature of ZnC_19_’s interaction, coupled with its significant conductivity change and visible colorimetric response, makes it a balanced candidate for dual-mode (electrochemical and colorimetric) sensing platforms where both sensitivity and reusability are desired.

#### NCI/ RDG analysis

**Fig. 10:**
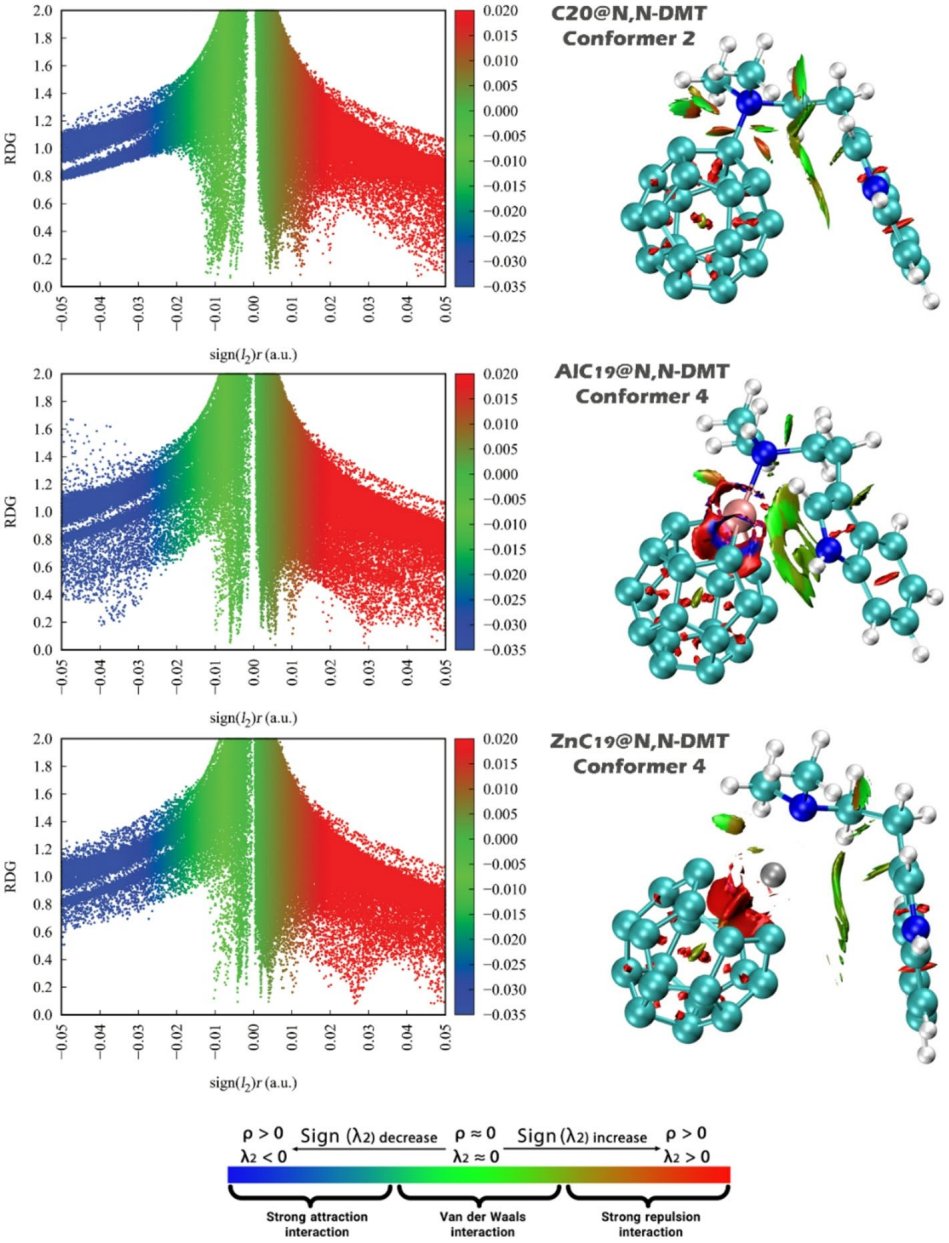
Non-Covalent Interaction (NCI) isosurfaces for C_20_@N, N-DMT, AlC_19_@N, N-DMT, and ZnC_19_@N, N-DMT. Green indicates van der Waals interactions, blue indicates strong attractive interactions, and red indicates steric repulsion.

Non-Covalent Interaction (NCI) analysis is essential in the design of complexes because it provides a detailed understanding of weak interactions (such as van der Waals forces, hydrogen bonding, and π-π stacking) that govern the stability, selectivity, and overall performance of sensor-analyte systems. These interactions, although individually weak, collectively influence the binding strength and orientation of the analyte on the sensor surface, directly affecting the sensor’s responsiveness and accuracy. Through computational methods, NCI analysis enables the visualization and quantification of these interactions by evaluating critical parameters such as the reduced density gradient (RDG), electron density (ρ), and the sign of the second eigenvalue of the electron density Hessian matrix [sign(λ_2_)ρ]. These parameters help distinguish between attractive, repulsive, and dispersive forces within the complex, offering deep insights into the nature and strength of the non-covalent interactions^65^. In this work, the NCI graphs for each of the designed complexes were systematically analyzed (Fig. 10).

The RDG and NCI analyses consistently show that all three systems (C_20_@N, N-DMT, AlC_19_@N, N-DMT, and ZnC_19_@N, N-DMT) exhibit a combination of attractive, van der Waals, and repulsive noncovalent interactions, but with clear quantitative and qualitative differences in their interaction strengths and stabilities. For C_20_@N, N-DMT (Conformer 2), the RDG versus sign(λ_2_)ρ plot spans approximately − 0.05 to + 0.05 a.u. along the horizontal axis and up to about 2.0 in RDG. The attractive region, appearing at negative sign(λ_2_)ρ values between roughly − 0.045 and − 0.020 a.u., shows minimum RDG values around 0.6–0.7, indicating moderate attractive interactions. The van der Waals region, centered near sign(λ_2_)ρ ≈ 0 and extending from about − 0.020 to + 0.005 a.u., reaches a minimum RDG of approximately 0.45 and represents the dominant interaction regime. Repulsive interactions appear at positive sign(λ_2_)ρ values above + 0.005 a.u., with minimum RDG values near 0.55 and a broad distribution up to higher RDG values, suggesting the presence of steric effects or less optimal overlap. The corresponding NCI isosurfaces show green regions associated with weakly attractive and van der Waals interactions, together with noticeable red regions, confirming that C_20_ binds reasonably well to N, N-DMT but with only moderate binding strength compared to the metal-containing systems.

In contrast, AlC_19_@N, N-DMT (Conformer 4) displays a much stronger and more favorable interaction profile. The RDG plot shows the attractive region extending to more negative sign(λ_2_)ρ values, approximately − 0.050 to -0.025 a.u., with minimum RDG values dropping to around 0.35. The van der Waals region, spanning roughly − 0.025 to + 0.005 a.u., reaches RDG values as low as about 0.30, while the repulsive region at positive sign(λ_2_)ρ values up to + 0.050 a.u. has minimum RDG values near 0.40. These lower RDG minima and more negative sign(λ_2_)ρ values quantitatively indicate significantly stronger noncovalent interactions than in the C_20_ system. The NCI plots support this interpretation by showing extensive green isosurfaces corresponding to strong attractive interactions, consistent with enhanced donor–acceptor interactions and higher second-order perturbation energies. The reduced extent of red regions further suggests fewer steric hindrances and more favorable molecular overlap, making the AlC_19_@N, N-DMT complex the most stable and robust among the three systems.

The ZnC_19_@N, N-DMT system (Conformer 5) exhibits an intermediate interaction strength between C_20_@N, N-DMT and AlC_19_@N, N-DMT. In this case, the attractive region in the RDG plot lies approximately between − 0.045 and − 0.020 a.u. in sign(λ_2_)ρ, with minimum RDG values around 0.40. The van der Waals region near zero sign(λ_2_)ρ reaches RDG values of about 0.35, while the repulsive region at positive sign(λ_2_)ρ values up to roughly + 0.045 a.u. shows minimum RDG values near 0.45. The NCI isosurfaces reveal a substantial amount of green regions indicative of stabilizing attractive interactions, though these are somewhat less intense and extensive than in the AlC_19_ system. Repulsive regions are relatively minor and comparable to those seen in AlC_19_@N, N-DMT, indicating a stable and well-aligned interaction geometry. Compared to C_20_@N, N-DMT, ZnC_19_@N, N-DMT clearly shows stronger attractive interactions and improved binding to N, N-DMT.

Overall, combining the qualitative NCI features with the quantitative RDG metrics, the interaction strength follows the order AlC_19_@N, N-DMT > ZnC_19_@N, N-DMT > C_20_@N, N-DMT. The progressive decrease in minimum RDG values in the attractive region (≈ 0.65 for C_20_, ≈ 0.40 for ZnC_19_, and ≈ 0.35 for AlC_19_) and the shift toward more negative sign(λ_2_)ρ values clearly demonstrate that metal incorporation, particularly Al, significantly enhances noncovalent interactions and overall complex stability with N, N-DMT.

#### Electrostatic potential (ESP) map

**Fig. 11:**
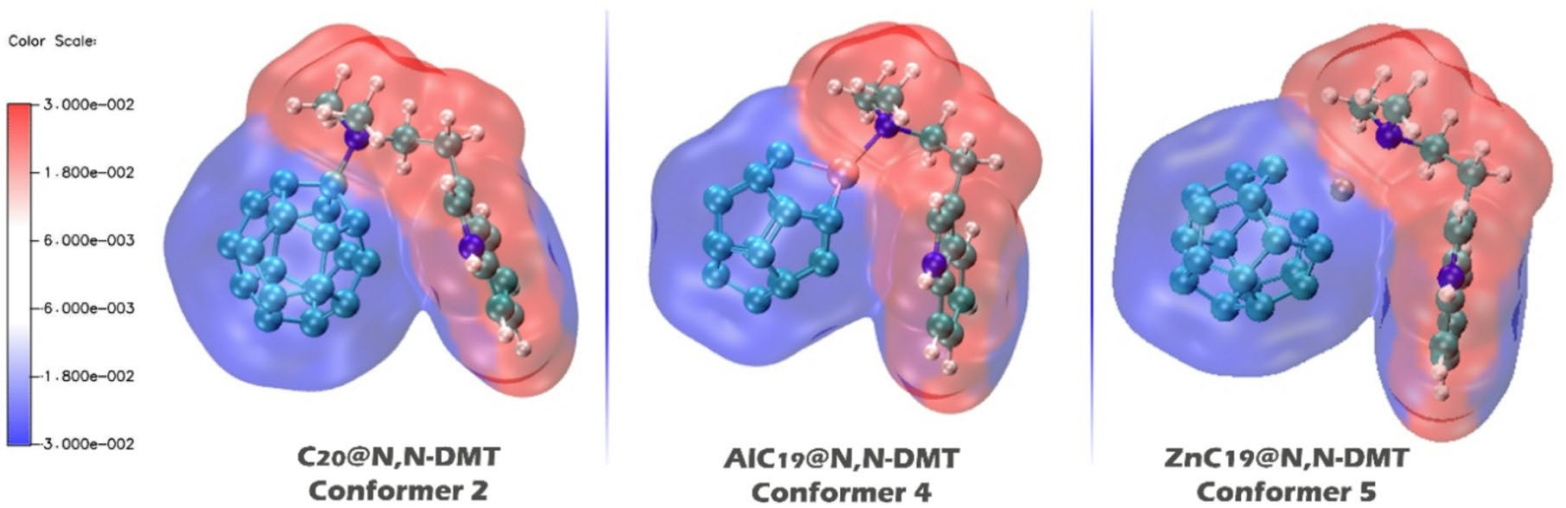
Electrostatic potential (ESP) contours for the complexes C_20_@N, N-DMT (Conformer 2), AlC_19_@N, N-DMT (Conformer 4), and ZnC_19_@N, N-DMT (Conformer 5), highlighting the electron density distribution and regions of charge transfer within each complex.

By including both MEP and electrostatic potential (ESP) sections, we aim to provide a comprehensive understanding of the electrostatic properties of the sensor materials and their potential for interaction with N, N-DMT. While the MEP gives a broader picture of electrostatic distribution, the ESP section offers a more focused analysis of molecular polarization and charge transfer, which are critical for understanding the stability and reactivity of the sensor-analyte complex. In ESP contours, red areas represent electron-rich regions, while blue areas represent electron-deficient regions, and the intensity of the colors indicates the strength of these interactions^66^. These contours are shown in Fig. 11.

For C_20_@N, N-DMT, the ESP contour shows a relatively balanced distribution of electrostatic potential. The red regions (indicating electron-rich areas) are observed around the aromatic regions of the complex, suggesting high electron density. The blue regions (indicating electron-deficient areas) are localized near the C_20_ core and the edges of the complex. This suggests that C_20_ has a relatively neutral electrostatic profile, with some electron-rich and electron-deficient regions that could interact with N, N-DMT, although the overall polarity is moderate. The distribution indicates that while C_20_ can interact with N, N-DMT, the interaction might not be as strongly polarized or directional compared to AlC_19_ or ZnC_19_. Also In the case of C_20_, which is initially non-polar, the adsorption of N, N-DMT induces a significant polarization, as seen in the ESP map, with distinct electron-rich (red) and electron-deficient (blue) regions. This results in a significant increase in dipole moment from 0.0 to 16.2 Debye.

For AlC_19_@N, N-DMT, the ESP contour reveals a more polarized structure. The red (electron-rich) regions are more pronounced, particularly around the ligand’s oxygen and nitrogen atoms, indicating regions of high electron density. The blue regions (electron-deficient areas) are less widespread but are noticeable around the central aluminum atom, which is less electron-rich compared to the surrounding atoms. This suggests that AlC_19_ is highly polarized with a stronger dipole moment, making it more capable of interacting with other molecules or ions that have complementary electrostatic properties. The stronger polarization and greater contrast between electron-rich and electron-deficient areas indicate that AlC_19_ may form stronger and more directed interactions with N, N-DMT compared to C_20_. For AlC_19_, which has an inherent dipole moment of 6.7 Debye, the interaction with N, N-DMT further polarizes the molecule, as shown by a stronger contrast in the ESP map and a dipole moment increase to 17.5 Debye.

For ZnC_19_@N, N-DMT, the ESP contour displays a significant contrast between electron-rich (red) and electron-deficient (blue) regions, similar to AlC_19_, but with a more pronounced distribution of electron density around the ligand and the zinc center. The electron-rich regions extend more widely, indicating stronger electron-donating interactions. The blue areas, though still present, are not as extensive as in C_20_, and they are concentrated around the zinc center. This suggests that ZnC_19_ exhibits a more pronounced electrostatic interaction with N, N-DMT, with a stronger dipole moment and more extensive electron density. ZnC_19_, initially more polarized (9.2 Debye), also becomes more polarized upon adsorption of N, N-DMT, but the change is less pronounced compared to AlC_19_, with a dipole moment increase to 13.9 Debye. The distribution suggests that ZnC_19_ is more highly polarized than C_20_ but potentially less than AlC_19_, making it highly capable of interacting with N, N-DMT but with a slightly less pronounced dipole than AlC_19_.

In the present study, the ESP contours and the ECT values are aligned so that regions of high electron density (red regions in the ESP map) correspond to electron donation from N, N-DMT to the sensor. This is supported by the negative ECT values, which indicate electron transfer from N, N-DMT to the sensor (C_20_, AlC_19_, or ZnC_19_). Conversely, the blue areas in the ESP maps, which represent electron-deficient regions in the sensor molecules, indicate that electron-deficient metal centers (Al or Zn) are accepting electrons from N, N-DMT.

The overlap of these two parameters reinforces the understanding that the polarization and electrostatic distribution observed in the ESP maps directly influence the electron donation and acceptance behaviors seen in the ECT analysis. For example, in AlC_19_ and ZnC_19_, the strong polarization indicated by the ESP maps corresponds to significant electron donation from N, N-DMT, as reflected by the more negative ECT values, demonstrating that the electrostatic complementarity between the donor (N, N-DMT) and the sensor (AlC_19_ or ZnC_19_) facilitates effective charge transfer.

Also, in all three complexes, the direction of charge transfer is from the electron-rich donor (i.e., analyte (N, N-DMT)) to the electron-deficient metal center (i.e., sensor (C_20_, AlC_19_, or ZnC_19_)), which overlaps with the values ​​reported for ECT (in Table 2).

**Table 7:** Dipole moment values ​​(in Debye) for the sensors in the neutral state (C20, AlC19, and ZnC19) and bound to N, N-DMT, showing polarization changes upon complex formation.

Structure	Dipole moment (Debye)
C_20_	0.0
AlC_19_	6.7
ZnC_19_	9.2
C_20_@N, N-DMT (Conformer 2)	16.2
AlC_19_@ N, N-DMT (Conformer 4)	17.5
ZnC_19_@ N, N-DMT (Conformer 5)	13.9

The analysis of the Electrostatic Potential (ESP) contours is further justified by the dipole moment values reported in Table 7. The dipole moment is a key indicator of the polarity of a molecule, with higher values indicating stronger polarization and a more pronounced separation of charge. These values provide additional support for the interpretation of the ESP data, confirming the relative strength of interactions and polarization in each of the complexes.

For C_20_, the dipole moment in its neutral form is 0.0 Debye, indicating that it is a non-polar molecule. After interacting with N, N-DMT, the dipole moment increases significantly to 16.2 Debye, suggesting that the binding event induces substantial polarization in the complex. This is in line with the ESP contour for C_20_@N, N-DMT, which showed an increased contrast between electron-rich (red) and electron-deficient (blue) regions. The ESP contour demonstrated noticeable electron density around the aromatic regions, which corresponds well with the increase in dipole moment, indicating that the interaction with N, N-DMT creates a strong dipole, enhancing its ability to interact with other species. Also, In the case of C_20_, which is initially non-polar, the adsorption of N, N-DMT induces a significant polarization, as seen in the ESP map, with distinct electron-rich (red) and electron-deficient (blue) regions. This results in a large increase in dipole moment from 0.0 to 16.2 Debye.

In its neutral form, AlC_19_ has a dipole moment of 6.7 Debye, which already indicates a moderately polarized structure. Upon interaction with N, N-DMT, the dipole moment increases to 17.5 Debye, the highest value among the three complexes. This increase is consistent with the ESP contour, which showed AlC_19_@N, N-DMT to be highly polarized, with a significant contrast between electron-rich and electron-deficient regions. The increased dipole moment and the corresponding ESP contour confirm that AlC_19_ forms the strongest dipole upon binding with N, N-DMT, indicating a stronger interaction due to the more pronounced electron density shifts, particularly around the aluminum center. For AlC_19_, which has an inherent dipole moment of 6.7 Debye, the interaction with N, N-DMT further polarizes the molecule, as shown by a stronger contrast in the ESP map and a dipole moment increase to 17.5 Debye.

For ZnC_19_, the neutral complex has a dipole moment of 9.2 Debye, which is higher than that of C_20_ and AlC_19_, indicating a moderately strong inherent polarization. This is reflected in the ESP contour, which shows a well-defined distribution of electron density, with red (electron-rich) and blue (electron-deficient) regions, suggesting a significant, though less intense, polarization compared to AlC_19_. Upon binding with N, N-DMT, the dipole moment increases to 13.9 Debye, a substantial increase but lower than the dipole moment increase seen in AlC19. This aligns with the ESP contours, which show significant electron density around the ligand and zinc center but with less pronounced polarization than in AlC_19_@N, N-DMT. The ZnC_19_@N, N-DMT complex, therefore, exhibits strong but moderate polarization, which is more than C_20_ but less than AlC_19_. ZnC_19_, initially more polarized (9.2 Debye), also becomes more polarized upon adsorption of N, N-DMT, but the change is less pronounced compared to AlC_19_, with a dipole moment increase to 13.9 Debye.

Thus, ZnC_19_@N, N-DMT lies between C_20_@N, N-DMT and AlC_19_@N, N-DMT in terms of interaction strength, exhibiting stronger polarization than C_20_ but weaker than AlC_19_, making it the most suitable for applications that require strong but not extreme polarization. These results are consistent with the energy values ​​reported in Table 3.

### Comparison with other literature

Comparing adsorption energies, band gaps, and recovery times across various studies reveals distinct strengths and limitations. Jalali Sarvestani et al. reported a relatively weak adsorption energy for amitriptyline (-16.1 kcal/mol) and a high HOMO-LUMO gap (5.18 eV), indicating moderate sensing capability but fast recovery, unsuitable for long-term retention. Ajdari et al. reported a stronger adsorption energy (-62.31 kcal/mol) for nortriptyline and a larger gap (7.73 eV), but with an extremely long recovery time (5.24 × 10^33^ s), making it ideal for adsorption rather than real-time sensing. Khalaj Zeighami et al. reported a powerful adsorption energy (-107.05 kcal/mol) for ethyl butyrate. Although their band gap was moderate (2.88 eV), the very long recovery time (3.47 × 10^66^ s) limits its use for sensing applications. In comparison, ZnC_19_ in this work shows a balanced performance with an adsorption energy of -22.6 kcal/mol and a HOMO-LUMO gap of 2.78 eV, which is favorable for electrochemical sensing. Its recovery time (2.28 × 10^24^ s) is longer than ideal but still acceptable for real-time detection, with a noticeable conductivity shift, making it suitable for both electrochemical and colorimetric sensing (Table 8). AlC_19_, with a stronger adsorption energy (-49.6 kcal/mol) and a slightly higher gap (2.97 eV), shows minimal conductivity change and an extremely long recovery time (3.70 × 10^4^ s), positioning it as a better adsorbent than a sensor (Table 8).

**Table 8:** Comparison of the results obtained in this work with other literature.

Study	Target analyte	Eads (kcal/mol)	HLG (eV)	τ (s)
Jalali Sarvestani et al.^67^	Amitriptyline	-16.1	5.18	6.51 × 10^− 1^
Ajdari et al.^13^	Nortriptyline	-62.31	7.73	5.24 × 10^33^
Khalaj Zeighami & Abdolkhani^14^	Ethyl butyrate	-107.05	2.88	3.47 × 10^66^
ZnC_19_ (This work)	N, N-DMT	-22.6	2.78	2.28 × 10^24^
AlC_19_ (This work)	N, N-DMT	-49.6	2.97	3.70 × 10^4^

## Conclusion

Based on a comprehensive computational investigation utilizing density functional theory (DFT), time-dependent DFT (TD-DFT), and non-covalent interaction (NCI) analysis, this study successfully evaluated the potential of pristine and doped C_20_ fullerenes (AlC_19_ and ZnC_19_) as sensing and adsorption platforms for N, N-Dimethyltryptamine (N, N-DMT).

The structural analysis indicated that doping significantly affects the molecular geometry, where ZnC_19_ has the most significant bond angle contraction (∼81.6°) and an elongation of the metal-carbon bond lengths (2.06 Å). The cohesive energy calculations showed that Zn doping also increases the structural stability (-198 kcal/mol) relative to Al doping, which destabilizes the system slightly (-179 kcal/mol) compared to pristine C_20_ (-186 kcal/mol). Electronically, the Molecular Electrostatic Potential (MEP) maps of the Al and Zn doped complexes show electron-deficient areas centered around the dopants as the sites to be most reactive to the electron-rich nucleobases (N, N-DMT). The frontier orbital analysis generally shows that doping increases the HOMO-LUMO gap (HLG), while ZnC_19_ has the largest gap of all complexes at 3.04 eV, which indicates electronic stability. However, after DMT adsorption, the amount of electrical conductivity change is most significant for ZnC_19_, dropping from 2.67 × 10^9^ to 1.67 × 10^9^ S/m (Sect.  3.3.1), which is an important characteristic for an electrochemical sensor. As for the adsorption energy (E_ads_), the strongest binding of AlC_19_@DMT (-49.57 kcal/mol) suggests the best possible configuration for DMT capture (Sect.  3.3.1), but the binding energy leads to an impracticably long recovery time for DMT of about 10^24^ s which limits reusability as a sensor.

For colorimetric applications, the UV-Vis analysis was crucial. ZnC_19_ showed the largest redshift in its maximum absorption wavelength (λ_max_) when it bound with DMT, moving from 455 nm to 523 nm. This change of 68 nm means a visible color shift from blue to green. This shift is much more noticeable than what was seen for C_20_, which had a 65 nm shift from 363 nm to 428 nm, at the edge of the visible spectrum. AlC_19_ had a small shift of only 11 nm.

The Natural Bond Orbital (NBO) analysis confirmed strong charge transfer interactions in all complexes. ZnC_19_ showed the highest second-order perturbation energy (E²) of 41.85 kcal/mol. The Non-Covalent Interaction (NCI) and Electrostatic Potential (ESP) analyses supported these findings. They showed that ZnC_19_@DMT has a balanced and favorable mix of attractive non-covalent interactions and a significant dipole moment of 13.9 Debye. This is beneficial for both sensing and a measurable optical response.

Finally, when comparing all the calculated parameters, such as structural stability, electronic properties like HLG and conductivity change, adsorption strength, recovery time, and optical response, ZnC_19_ stands out as the best dual-function material. ZnC_19_ is the strongest candidate for both an electrochemical sensor because of its significant conductivity change (Δσ = 1.00 × 10^9^ S/m) and acceptable recovery time of about 10 h. It also serves well as a colorimetric sensor due to its large, easily noticeable redshift in absorption (Δλ_max_ = 68 nm). In contrast, AlC_19_ is the top choice for N, N-DMT removal applications because of its very strong adsorption energy (-49.57 kcal/mol) and effectively irreversible binding. Lastly, the promising theoretical data mentioned here strongly suggest the need for further experimental validation to evaluate the practical usefulness, sensitivity, and selectivity of the ZnC_19_ and AlC_19_ systems in real-world settings.

It is important to note that this study was conducted entirely using computational methods. A more accurate and comprehensive evaluation of these findings will require experimental validation. Future studies should aim to synthesize the ZnC_19_ sensor and conduct electrochemical and colorimetric tests to verify the computational predictions. To experimentally validate the promising computational results presented here, several steps could be taken. First, the ZnC_19_ sensor could be synthesized using standard methods for doping fullerenes, followed by characterization using techniques such as scanning electron microscopy (SEM) and X-ray diffraction (XRD) to confirm the structural integrity and doping. Next, electrochemical characterization could be performed using cyclic voltammetry (CV) to assess the sensor’s electrochemical response to N, N-DMT under different concentrations. For further validation, a colorimetric assay could be conducted, utilizing UV-vis spectroscopy to measure the wavelength shift associated with N, N-DMT binding. Experimental investigations will help to validate the sensor performance in real-world conditions and provide a deeper understanding of its practical application for N, N-DMT detection in different environments. It is worth noting that expanding the study to include the detection of additional psychoactive substances or biomarkers could pave the way for the development of versatile, high-performance sensor platforms for medical diagnostics, forensic analysis, and drug monitoring.

## Data Availability

All data generated or analyzed during this study are included in this published article.
